# Intestinal FGF15 regulates bile acid and cholesterol metabolism but not glucose and energy balance

**DOI:** 10.1172/jci.insight.174164

**Published:** 2024-04-08

**Authors:** Nadejda Bozadjieva-Kramer, Jae Hoon Shin, Ziru Li, Alan C. Rupp, Nicole Miller, Stace Kernodle, Nicolas Lanthier, Paulina Henry, Nikhil Seshadri, Andriy Myronovych, Ormond A. MacDougald, Robert W. O’Rourke, Rohit Kohli, Charles F. Burant, Amy E. Rothberg, Randy J. Seeley

**Affiliations:** 1 Research Service, Veterans Affairs Ann Arbor Healthcare System, Ann Arbor, Michigan, USA.; 2 Department of Surgery and; 3 Molecular and Integrative Physiology, University of Michigan, Ann Arbor, Michigan, USA.; 4 Center for Molecular Medicine, MaineHealth Institute for Research, Scarborough, Maine, USA.; 5 Division of Metabolism, Endocrinology and Diabetes, Department of Internal Medicine, University of Michigan, Ann Arbor, Michigan, USA.; 6 Hepato-Gastroenterology Department, Saint-Luc University Clinics, and; 7 Laboratory of Hepatology and Gastroenterology, Institute of Experimental and Clinical Research, UCLouvain, Brussels, Belgium.; 8 Pathological Anatomy Department, Institute of Pathology and Genetics, Gosselies, Belgium.; 9 Division of Gastroenterology, Hepatology and Nutrition, Children’s Hospital Los Angeles, Los Angeles, California, USA.

**Keywords:** Gastroenterology, Metabolism, Cholesterol, Glucose metabolism, Obesity

## Abstract

Fibroblast growth factor 15/19 (FGF15/19, mouse/human ortholog) is expressed in the ileal enterocytes of the small intestine and released postprandially in response to bile acid absorption. Previous reports of *FGF15^–/–^* mice have limited our understanding of gut-specific FGF15’s role in metabolism. Therefore, we studied the role of endogenous gut-derived FGF15 in bile acid, cholesterol, glucose, and energy balance. We found that circulating levels of FGF19 were reduced in individuals with obesity and comorbidities, such as type 2 diabetes and metabolic dysfunction–associated fatty liver disease. Gene expression analysis of ileal FGF15-positive cells revealed differential expression during the obesogenic state. We fed standard chow or a high-fat metabolic dysfunction-associated steatohepatitis–inducing diet to control and intestine-derived FGF15-knockout (FGF15^INT-KO^) mice. Control and FGF15^INT-KO^ mice gained similar body weight and adiposity and did not show genotype-specific differences in glucose, mixed meal, pyruvate, and glycerol tolerance. FGF15^INT-KO^ mice had increased systemic bile acid levels but decreased cholesterol levels, pointing to a primary role for gut-derived FGF15 in regulating bile acid and cholesterol metabolism when exposed to obesogenic diet. These studies show that intestinal FGF15 plays a specific role in bile acid and cholesterol metabolism regulation but is not essential for energy and glucose balance.

## Abstract 2

Intestinal FGF15 plays a specific and essential role in bile acid and cholesterol metabolism regulation but is not necessary for the control of energy and glucose balance.

## Graphical abstract

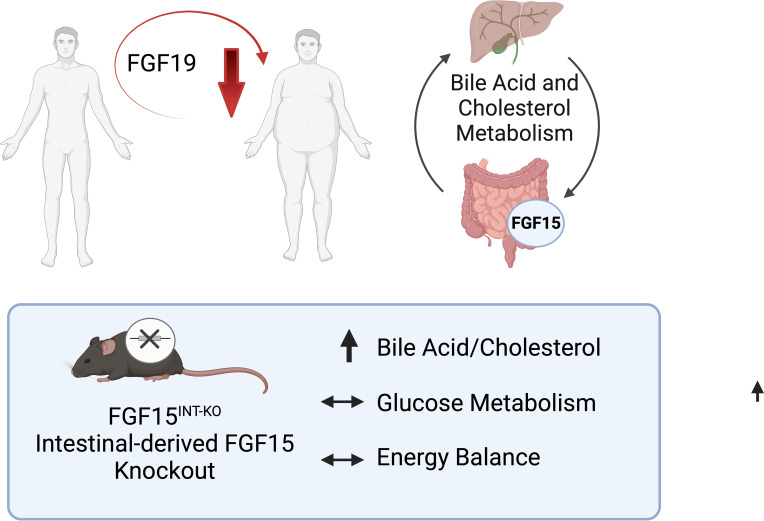

## Introduction

The increasing prevalence of obesity and its associated comorbid complications of type 2 diabetes, metabolic dysfunction–associated fatty liver, and cardiovascular morbidity is a global health concern (1, 2). Western lifestyle, including high-calorie intake and sedentary behavior, likely contributes to obesity and obesity-related disorders. The consumption of high-calorie foods, including high fructose content, has been shown to disrupt gastrointestinal (GI) and hepatic function, leading to impaired GI barrier function and metabolic dysfunction–associated steatotic liver disease (MASLD) characterized by hepatic steatosis and possible further progression into metabolic dysfunction–associated steatohepatitis (MASH). Additionally, the consumption of high-fat foods alters enterohepatic circulation, increasing bile acid levels and affecting bile acid composition, which in turn can have potent effects on the gut microbiome (3). These alterations highlight the gut/liver axis as an essential link in the development of obesity-related hepatic lipid disorders.

High-fat diet–induced (HFD-induced) obesity results in cholesterol accumulation that likely contributes to lipid disorders. Cholesterol and bile acid synthesis are closely linked, as primary bile acids are synthesized from cholesterol in the liver. The past 3 decades have witnessed explosive growth in our understanding of bile acids as signaling molecules in metabolism (4–6). Bile acids are physical detergents that are essential for the intestinal absorption of lipids and vitamins and can increase cholesterol solubility to promote the intestinal absorption of cholesterol. Beyond their traditional role as surfactants, a considerable range of evidence points toward bile acids acting as hormones by interacting with 2 receptors: the cell surface receptor TGR5 (encoded by *GPBAR1*) and the nuclear ligand-activated nuclear receptor Farnesoid X receptor (FXR) (reviewed in refs. 4–6). Bile acids are absorbed in the intestinal lumen and activate intestinal FXR and its downstream target fibroblast growth factor 15/19, where FGF15 is the mouse ortholog and FGF19 the human ortholog. FGF15 is expressed in ileal enterocytes of the small intestine and released postprandially in response to gut bile acid absorption (7–9). Bile acids act to induce FGF15/19 secretion and regulate diverse aspects of hepatic metabolism, including cholesterol catabolism (10). FGF15/19 provides negative feedback that reduces subsequent bile acid secretion from the liver and the gallbladder (11, 12). Therefore, bile acids and FGF15/19 both act as negative feedback signals to regulate bile acids and cholesterol synthesis.

FGF15/19 has been widely hypothesized to act as a gut hormone that serves a critical function in the gut/liver axis and potentially in other organ systems as well (9). Testing this hypothesis has been complicated by several issues. As its name implies, FGF15/19 is a growth factor and is expressed by several tissues during development (13–15). Consequently, whole-body FGF15-knockout mice have diverse developmental issues that make assessing the role of FGF15 in adult animals exceptionally difficult. Hence, much of what we know about function comes from FGF15/19 infusion experiments (16–21). Although such experiments can reveal the potential actions of FGF15/19 and its therapeutic potential, it is impossible to match infusions to normal circulating levels of FGF15, particularly since reliable assays for FGF15 have been difficult to develop (22, 23). To this end, we developed a mouse model that allows us to delete FGF15 specifically from the intestine in the adult animal (24). Tissue-specific loss-of-function experiments in adult animals are essential to fully assess the various hypothesized roles of intestinally derived FGF15. This is particularly important because FGF15 was originally thought to be secreted solely by the gut. However, recent studies have identified a source of FGF15 in the central nervous system, specifically within the dorsal medial hypothalamus (DMH) (25–27). Data from these studies show that central FGF15-expressing neurons in the DMH control glucagon secretion and hepatic gluconeogenesis (25, 26). However, the gut-specific contribution of FGF15/19 to these metabolic benefits remains unclear.

Several reports have shown that circulating FGF19 levels are lower in individuals with obesity (28–31) and MASLD (32, 33) supporting the role of FGF19 as a hormone in metabolism. We and others have shown that pharmacologically increasing FGF15/19 levels in rodent models of metabolic disease results in multiple metabolic benefits, including increased energy expenditure, reduced adiposity, and improved lipid and glucose homeostasis (16–21). Notably, FGF19 levels rise 2–4 hours postprandially, which is considerably later than the postprandial rise of insulin and glucagon-like peptide 1 (GLP-1) (34). The metabolic advantages of this delayed postprandial increase of FGF15/19 and the elimination of its potential mitogenic activity by protein engineering has made FGF19 an attractive candidate for the treatment of metabolic and hepatic lipid disorders (35–38).

The goal of our study was to dissect the specific role of intestine-derived FGF15 in energy balance, glucose metabolism, and hepatic lipid metabolism. We used gut-derived FGF15-knockout mice (FGF15^INT-KO^) and littermate controls on standard chow diet and Western diet (diet-induced obesity MASH [DIO-MASH] diet composed of fat, fructose, and cholesterol). Our findings show that intestinal FGF15 is a major regulator of bile acid synthesis, and FGF15^INT-KO^ mice had higher plasma, hepatic, and cecal content bile acid levels compared with controls under standard chow and DIO-MASH diets. However, our data also show that gut-derived FGF15 is not essential to the regulation of energy balance and glucose metabolism during standard and obesogenic diet. This highlights the tissue-specific role of intestinal FGF15 in bile acid and lipid metabolism but not glucose metabolism or energy balance.

## Results

### Circulating FGF19 levels decrease in human participants with obesity and comorbidities.

**Figure 1:**
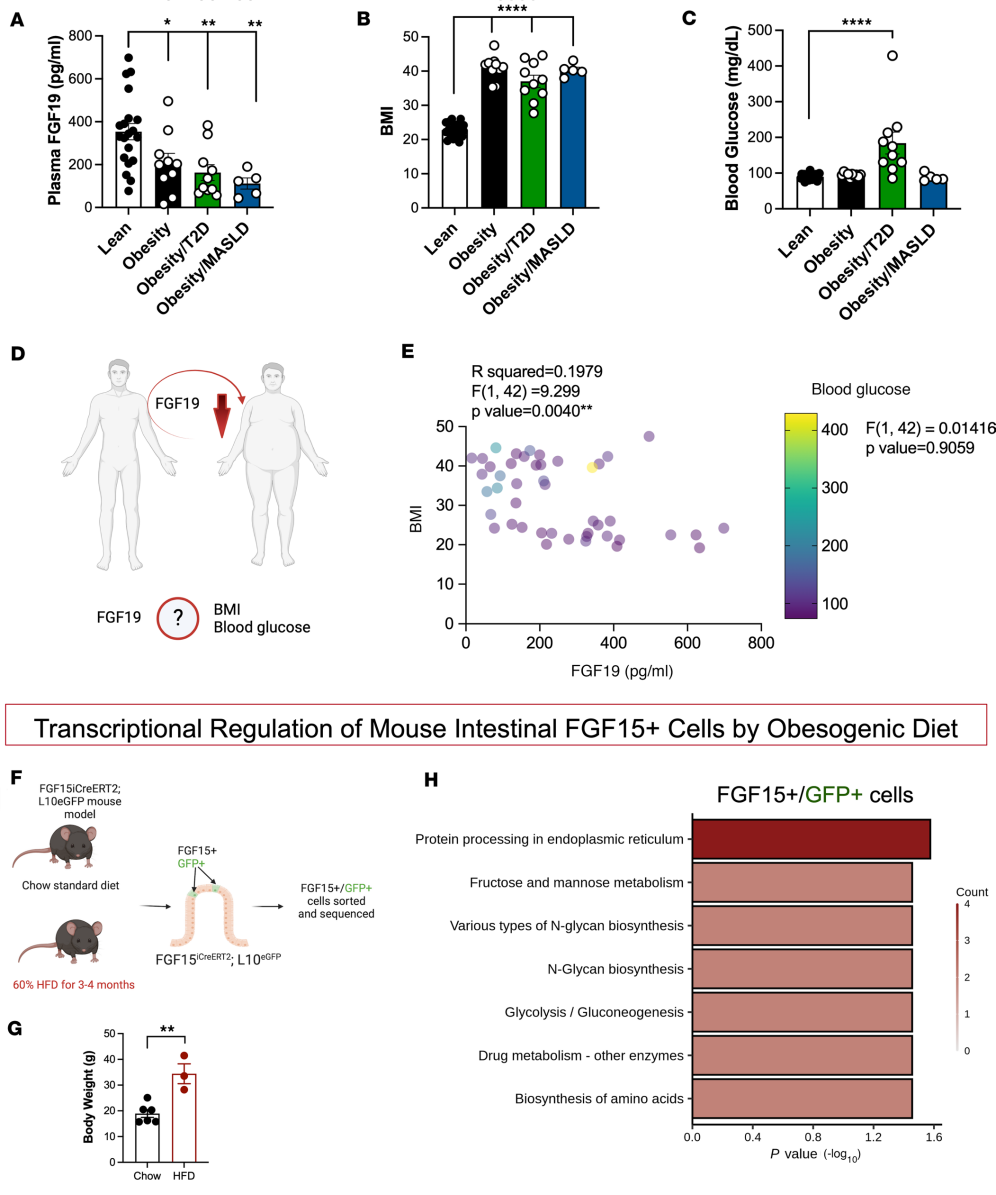
Circulating FGF19 levels decrease in human participants with obesity and comorbidities. (**A**) Postprandial (180 minutes after mixed meal) levels of circulating FGF19. (**B**) BMI. (**C**) Postprandial (180 minutes after mixed meal) glucose levels. (**D**) We asked whether postprandial circulating FGF19 levels correlate with BMI and/or blood glucose levels in these human individuals. (**E**) Multiple linear regression of FGF19 levels, blood glucose, and BMI (*n* = 20 Lean, *n* = 10 Obese, *n* = 10 Obese/T2D, *n* = 5 Obese/MASLD humans). (**F**) RNA-Seq was used to determine the transcriptional effect of obesogenic state/diet in ileal FGF15-expressing cells using FGF15^iCreERTM2^ mice crossed to the L10^eGFP^ reporter. (**G**) Body weight of standard chow–fed (*n* = 6) and high-fat diet–fed (HFD-fed) (*n* = 3) FGF15iCreERT2 L10eGFP mice. (**H**) KEGG pathway analysis of differential gene expression of intestinal FGF15^+^ cells in standard chow–fed (*n* = 6) and HFD-fed (*n* = 3) FGF15iCreERT2 L10eGFP mice. Data for **A**–**C** are shown as means ± SEM. *P < 0.05, 1-way ANOVA (Dunnett’s multiple comparisons test). Data for **E** are shown as multiple linear regression estimating the relationship of postprandial circulating FGF19 with BMI and blood glucose. Data for **G** are shown as means ± SEM. *P < 0.05, 2-tailed Student’s *t* test (unpaired). Data for **H** are shown as KEGG pathway analysis of RNA-Seq data of ileum FGF15^+^ cells in chow and HFD-fed FGF15iCreERT2 L10eGFP mice.

Previous reports have shown that circulating FGF19 levels are reduced in individuals with metabolic disorders and MASLD (30, 39). Consistent with these published findings, our data also showed that postprandial FGF19 concentrations (180 minutes after mixed meal) decreased in people with obesity (average BMI = 41), patients with obesity and T2D (average BMI = 39), and patients with obesity and MASLD (no T2D) (average BMI = 40) compared with lean people (average BMI = 23.5) (Figure 1A). BMI was significantly higher in people with obesity, obesity and T2D, obesity and MASLD compared with control lean people (Figure 1B). However, postprandial blood glucose was higher only in patients with obesity and T2D compared with lean controls (Figure 1C). We asked whether postprandial circulating FGF19 levels correlate with BMI and/or blood glucose levels in these populations (Figure 1D). Multiple linear regression analysis of FGF19 levels with blood glucose and BMI as independent variables showed that postprandial circulating FGF19 levels significantly correlated with BMI but not with postprandial blood glucose levels (Figure 1E).

### DIO leads to transcriptional changes in ileal FGF15-expressing enterocytes.

Next, we wanted to identify the transcriptional changes that occur in FGF15/19-expressing cells with obesity. We used RNA-Seq to determine the transcriptional effect of obesogenic state/diet specifically in ileal FGF15-expressing cells using FGF15^iCreERTM2^ mice crossed to the L10^eGFP^ reporter (Figure 1F). These mice were fed either standard chow or 60% HFD (Research Diets) for 3–4 months (Figure 1G). Kyoto Encyclopedia of Genes and Genomes (KEGG) pathway analysis identified the top pathways differentiated in FGF15-expressing cells between chow-fed and HFD-fed mice, including protein processing, fructose and mannose metabolism, and glycolysis/gluconeogenesis (Figure 1H).

### Intestine-derived FGF15 is not required for energy balance and glucose metabolism under standard chow diet.

**Figure 2:**
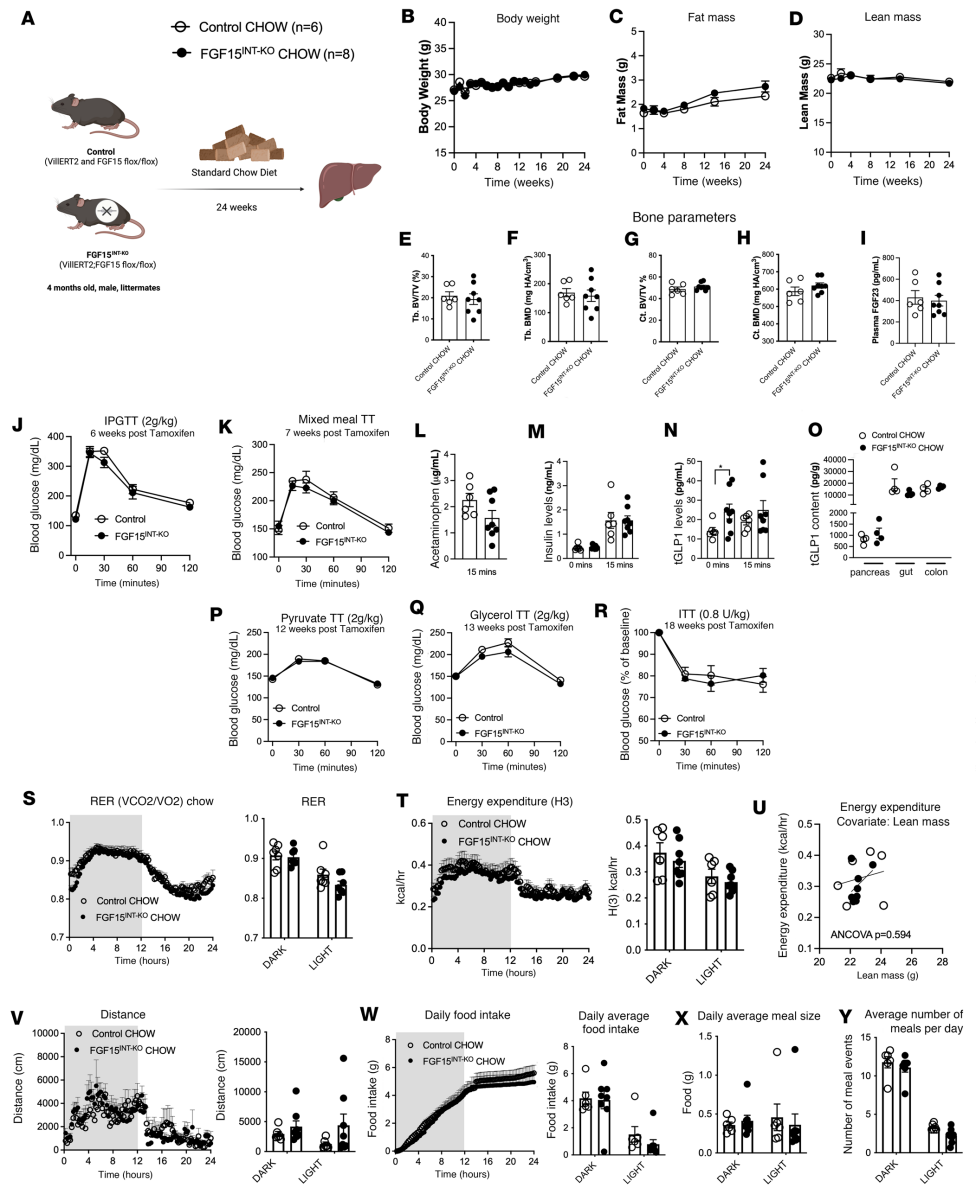
Intestine-derived FGF15 is not required for energy balance and glucose metabolism under standard chow diet. (**A**) Experimental timeline of control and FGF15^INT-KO^ mice fed standard chow diet. (**B**) Longitudinal body weight, (**C**) fat mass, and (**D**) lean mass. Bone parameters, including (**E**) trabecular bone volume fraction (Tb. BV/TV), (**F**) trabecular bone mineral density (Tb. BMD), (**G**) cortical bone area (Ct. BV/TV), (**H**) cortical bone mineral density (Ct. BMD). (**I**) Circulating FGF23 levels. (**J**) IPGTT (2 g/kg). (**K**) Mixed meal tolerance test. (**L**) Gastric emptying rate measured by acetaminophen levels at 15 minutes after mixed meal. (**M**) Insulin levels at baseline (4 hours fast) and 15 minutes after mixed meal. (**N**) Total GLP-1 levels at baseline (4 hours fast) and 15 minutes after mixed meal. (**O**) GLP-1 levels in pancreas, small intestine, and colon. (**P**) Pyruvate tolerance test (2 g/kg). (**Q**) Glycerol tolerance test (2 g/kg). (**R**) Insulin tolerance test (0.8 U/kg). Indirect colometry measurements averaged for 3 days. (**S**) Respiratory exchange ratio (RER). (**T**) Energy expenditure H(3). (**U**) ANCOVA for energy expenditure with lean mass as covariate. (**V**) Distance/locomotor activity. (**W**) Daily food intake. (**X**) Daily average meal size. (**Y**) Average number of meals per day. Animal numbers for **A**–**N**, **P**–**R**, and **T**–**Y** are control (*n* = 6), FGF15^INT-KO^ (*n* = 8). Animal numbers for **O** are control (*n* = 4), FGF15^INT-KO^ (*n* = 4). Animal numbers for **S** are control (*n* = 6), FGF15^INT-KO^ (*n* = 7). Data are shown as means ± SEM. *P < 0.05, 2-tailed Student’s *t* test (unpaired) comparing responses between genotypes. VCO2/VO2, carbon dioxide output/oxygen consumption.

FGF15^INT-KO^ and control mice received tamoxifen (150 mg/kg, 3 doses, 48 hours apart) and were maintained on standard chow diet (Figure 2A). Body weight, fat mass, and lean mass were comparable between the 2 genotypes (Figure 2, B–D). Additionally, we did not observe any difference in bone parameters, including trabecular bone volume fraction (Tb. BV/TV), bone mineral density (Tb. BMD), cortical bone volume fraction (Ct. BV/TV), and bone mineral density (Ct. BMD) (Figure 2, E–H). Fibroblast growth factor 23 (FGF23), the third member of the endocrine FGFs along with FGF15/19 and FGF21, is a bone-derived hormone that regulates mineral homeostasis and consequently bone mineral density (40). The plasma concentrations of FGF23 were also comparable between the genotypes (Figure 2I). These data indicate FGF15 does not play a significant role in the gut/bone axis under standard diet conditions.

Intraperitoneal tolerance test (IPGTT, 2 g/kg) and a mixed meal tolerance test (MMTT, flat dose of 200 μL Ensure Plus spiked with 40 mg dextrose and 4 mg acetaminophen, MilliporeSigma) revealed no differences in glucose excursion between FGF15^INT-KO^ mice and controls (Figure 2, J and K). Gastric emptying and insulin concentration at fasting and 15 minutes after mixed meal were also not different (Figure 2, L and M). However, FGF15^INT-KO^ mice had higher fasting GLP-1 concentration but comparable postprandial GLP-1 response to that of the controls (Figure 2N). These differences were not associated with alterations in GLP-1 content of pancreas, small intestine, or colon (Figure 2O). Additionally, FGF15^INT-KO^ mice had preserved hepatic glucose production during pyruvate and glycerol tolerance tests (2 g/kg; Figure 2, P and Q), as well as blood glucose concentration during an insulin tolerance test (ITT, 0.8 U/kg; Figure 2R). Although the respiratory exchange ratio (RER) was slightly lower in FGF15^INT-KO^ mice compared with control, the average RER during the light and dark cycles was not significantly different (Figure 2S). Similarly, the energy expenditure and the covariate analysis adjusting for lean mass did not reveal differences in energy expenditure between the 2 genotypes (Figure 2, T and U). The locomotor activity, daily food intake, and daily meal size and number of meals were comparable between the 2 groups (Figure 2, V–Y). These data show that gut-derived FGF15 is not required for the maintenance of glucose metabolism and energy balance in mice fed a standard chow diet.

### Intestine-derived FGF15 is not required for energy balance and glucose metabolism when maintained on a DIO-MASH diet.

**Figure 3:**
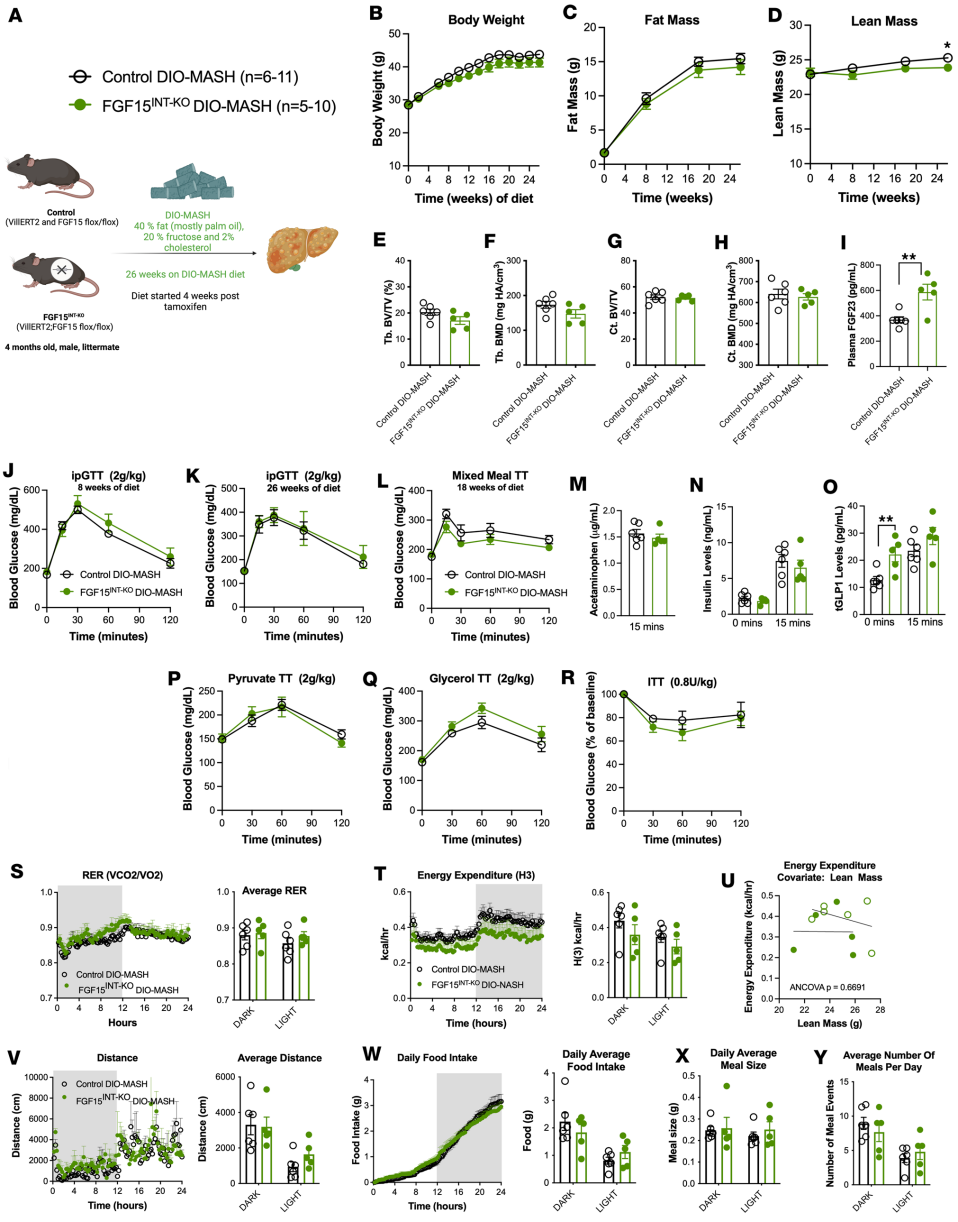
Intestine-derived FGF15 is not required for energy balance and glucose metabolism when maintained on a DIO-MASH diet. (**A**) Experimental timeline of control and FGF15^INT-KO^ mice fed DIO-MASH diet, composed of 40% fat (palm oil), 20% fructose, 2% cholesterol for 26 weeks. (**B**) Longitudinal body weight, (**C**) fat mass, and (**D**) lean mass. Bone parameters including (**E**) trabecular bone volume fraction (Tb. BV/TV), (**F**) trabecular bone mineral density (Tb. BMD), (**G**) cortical bone area (Ct. BV/TV), (**H**) cortical bone mineral density (Ct. BMD). (**I**) Circulating FGF23 levels. (**J**) IPGTT (2 g/kg) 8 weeks of diet. (**K**) IPGTT (2 g/kg) 26 weeks of diet. (**L**) Mixed meal tolerance test. (**M**) Gastric emptying rate measured by acetaminophen levels at 15 minutes after mixed meal. (**N**) Insulin levels at baseline (4 hours fast) and 15 minutes after mixed meal. (**O**) Total GLP-1 levels at baseline (4 hours fast) and 15 minutes after mixed meal. (**P**) Pyruvate tolerance test (2 g/kg). (**Q**) Glycerol tolerance test (2 g/kg). (**R**) Insulin tolerance test (0.8 U/kg). Indirect colometry measurements averaged for 3 days. (**S**) Respiratory exchange ratio (RER). (**T**) Energy expenditure H(3). (**U**) ANCOVA for energy expenditure with lean mass as covariate. (**V**) Distance/locomotor activity. (**W**) Daily food intake. (**X**) Daily average meal size. (**Y**) Average number of meals per day. Animal numbers for **B**–**D**, **P**, and **Q** are control (*n* = 11), FGF15^INT-KO^ (*n* = 10). Animal numbers for **E**–**J**, **L**–**O**, and **S**–**Y** are control (*n* = 6), FGF15^INT-KO^ (*n* = 5). Animal numbers for **K** and **R** are control (*n* = 5), FGF15^INT-KO^ (*n* = 5). Data are shown as means ± SEM. *P < 0.05, 2-tailed Student’s *t* test (unpaired) comparing responses between genotypes.

KEGG pathway analysis identified the top pathways different in FGF15-expressing cells between chow-fed and HFD-fed mice, including protein processing, fructose and mannose metabolism, and glycolysis/gluconeogenesis (Figure 1H). Next, we evaluated the role of intestinal FGF15 when challenged with hypercaloric diet on energy balance and glucose metabolism. FGF15^INT-KO^ and control mice received tamoxifen (150 mg/kg, 3 doses, 48 hours apart) and 4 weeks later were switched from standard chow diet to DIO-MASH diet, also known as GAN diet (40% fat, 20% fructose, 2% cholesterol; Research Diets Inc, catalog D09100310; Figure 3A). Body weight and fat mass were comparable between the 2 genotypes (Figure 3, B and C). The lean mass of FGF15^INT-KO^ mice was lower compared with controls at 26 weeks of DIO-MASH diet (Figure 3D). We did not observe any difference in bone parameters, including Tb. BV/TV, Tb. BMD, Ct. BV/TV, or Ct. BMD (Figure 3, E–H). However, the concentration of FGF23 was increased in FGF15^INT-KO^ mice compared with controls (Figure 3I). IPGTT (2 g/kg) performed at 8 and 26 weeks of diet revealed no difference in glucose excursion between FGF15^INT-KO^ mice and controls (Figure 3, J and K). Similarly, an MMTT also revealed no difference in glucose excursion between FGF15^INT-KO^ mice and controls (Figure 3L). Gastric emptying and insulin concentration at fasting and 15 minutes after mixed meal were not different between the 2 groups (Figure 3, M and N). However, FGF15^INT-KO^ mice fed DIO-MASH diet also had higher fasting GLP-1 concentration but were comparable to controls in postprandial GLP-1 response (Figure 3O). FGF15^INT-KO^ mice had preserved glucose response during pyruvate and glycerol tolerance tests (2 g/kg; Figure 3, P and Q) and ITT (0.8 U/kg; Figure 3R). The RER was similar in FGF15^INT-KO^ animals and controls (Figure 3S). Similarly, the energy expenditure and the covariate analysis taking lean mass into consideration did not reveal differences in energy expenditure between the 2 genotypes (Figure 3, T and U). The locomotor activity, daily food intake, daily meal size, and number of meals were comparable between the 2 groups (Figure 3, V–Y). These data showed that gut-derived FGF15 is not required for the maintenance of glucose metabolism or energy balance during DIO-MASH diet.

### Intestinal morphometry is not dependent on intestinal FGF15 expression.

There were no differences between FGF15^INT-KO^ and control mice in small bowel length, weight, and length/weight (Supplemental Figure 1, A–C; supplemental material available online with this article; https://doi.org/10.1172/jci.insight.174164DS1) nor large bowel length, weight, and length/weight (Supplemental Figure 1, D–F). Diet-dependent alterations showed lower small bowel length, and lower large bowel length and weight, in DIO-MASH mice compared with standard chow–fed mice. Analysis of ileum cross sections showed no difference in villi height, crypt depth, or ratio of villi height/crypt depth between the genotypes in chow-fed mice (Supplemental Figure 1, G–I). However, DIO-MASH FGF15^INT-KO^ mice trended toward decreased villi height compared with controls (Supplemental Figure 1G), with no difference in crypt depth or the ratio of villi height/crypt depth between the groups (Supplemental Figure 1, H and I).

### Intestinal FGF15 regulates enterohepatic bile acid metabolism.

**Figure 4:**
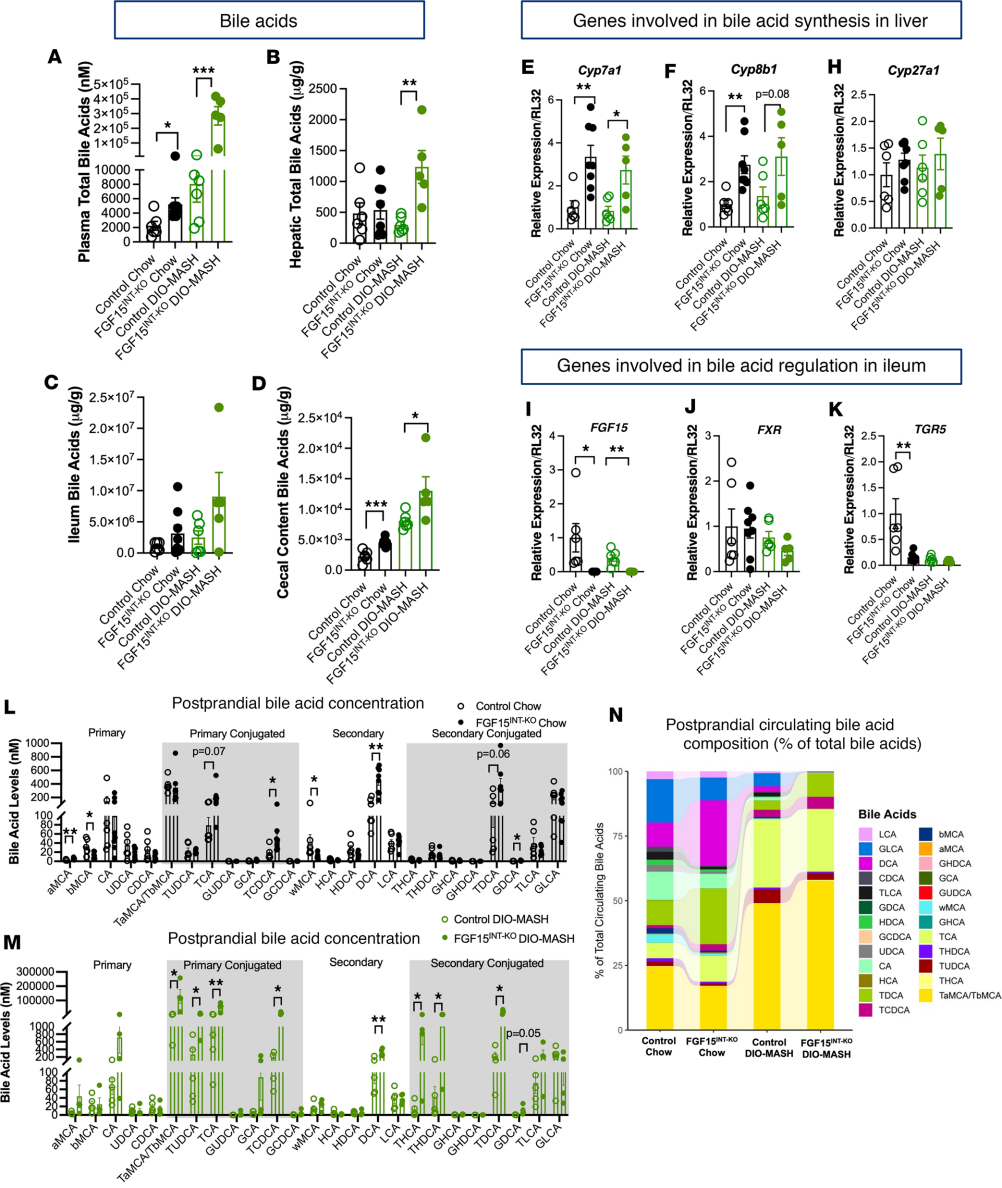
Intestinal FGF15 regulates tissue-specific bile acid levels and composition. (**A**) Plasma total bile acids (postprandial). (**B**) Hepatic total bile acids. (**C**) Ileum total bile acids. (**D**) Cecal contents total bile acids. RNA expression of genes involved in bile acid synthesis in the liver. (**E**) *Cyp7a1*. (**F**) *Cyp8b1*. (**G**) *Cyp27a1*. RNA expression of genes involved in bile acid regulation in the ileum. (**I**) *FGF15*. (**J**) *NR1H4* (FXR). (**K**) *GPBAR1* (TGR5). (**L**) Postprandial circulating bile acid composition levels in chow-fed control and FGF15^INT-KO^ mice. (**M**) Postprandial circulating bile acid composition levels in DIO-MASH–fed control and FGF15^INT-KO^ mice. (**N**) Postprandial circulating bile acid composition shown as percent (%) of total bile acid levels in chow and DIO-MASH-fed control and FGF15^INT-KO^ mice. Animal numbers for **A**–**J** are control chow (*n* = 6), FGF15^INT-KO^ chow (*n* = 8), control DIO-MASH (*n* = 6), FGF15^INT-KO^ DIO-MASH (*n* = 5). Animal numbers for **K** are control chow (*n* = 6), FGF15^INT-KO^ chow (*n* = 7), control DIO-MASH (*n* = 6), FGF15^INT-KO^ DIO-MASH (*n* = 5). Animal numbers for **L**–**N** are control chow (*n* = 6), FGF15^INT-KO^ chow (*n* = 8), control DIO-MASH (*n* = 5), FGF15^INT-KO^ DIO-MASH (*n* = 4). Data are shown as means ± SEM. *P < 0.05, **P < 0.01, and ***P < 0.001, 2-tailed Student’s *t* test (unpaired) comparing responses between genotypes per diet.

Plasma bile acid levels were elevated in both chow and DIO-MASH FGF15^INT-KO^ mice compared with controls (Figure 4A). Hepatic bile acid content was comparable between chow controls and FGF15^INT-KO^ mice (Figure 4B). When challenged with diet high in fat, hepatic bile acid content decreases, as seen in our control DIO-MASH mice (41). However, when fed DIO-MASH diet, the FGF15^INT-KO^ mice did not have this diet-dependent decrease in hepatic bile acid levels and thus had increased hepatic bile acid levels compared with controls (Figure 4B). There were no significant differences in ileal bile acid content (Figure 4C). The bile acid levels in the cecal content were increased in FGF15^INT-KO^ mice under standard chow and DIO-MASH diet (Figure 4D). Consistent with increased circulating bile acid levels, lack of intestinal FGF15 resulted in higher expression of the classic pathway bile acid synthesis genes cholesterol 7a-hydroxylase (*Cyp7a1*) and sterol 12-alpha-hydroxylase (*Cyp8b1*) in FGF15^INT-KO^ compared with control mice under both diets (Figure 4, E and F). There were no differences in the expression of the alternative pathway sterol 27-hydroxylase (*Cyp27a1*) between the genotypes (Figure 4H). Ileal FGF15 expression was not detected in FGF15^INT-KO^ mice, and the ileal expression of *FXR* was similar in control and FGF15^INT-KO^ mice (Figure 4, I and J). However, TGR5 expression was significantly lower in chow FGF15^INT-KO^ mice compared with chow controls, and TGR5 expression was very low in both DIO-MASH genotype groups (Figure 4K).

FGF15^INT-KO^ mice fed both standard chow and DIO-MASH diet had higher systemic bile acid levels compared with respective controls. Therefore, we analyzed the levels and composition of bile acid species in the 4 groups (Figure 4, L–N). Although the levels of the primary bile acid cholic acid (CA) were not different under either diet, FGF15^INT-KO^ mice had significantly higher levels of conjugated taurocholic acid (TCA), deconjugated secondary bile acid deoxycholic acid (DCA), and conjugated secondary taurodeoxycholic bile acid (TDCA) (Figure 4, L and M). Tauromuricholate (Tα/βMCA), tauroursodeoxycholic acid (TUDCA), taurohyocholic acid (THCA), and taurohyodeoxycholic acid (THDCA) were also higher in DIO-MASH FGF15^INT-KO^ mice compared with controls (Figure 4M). We analyzed the changes in bile acids as a percentage of total bile acids, which also showed diet- and genotype-dependent changes. Although DCA levels were elevated in FGF15^INT-KO^ mice in both diets, the percentage of DCA as part of total bile acids appeared substantially lower in DIO-MASH mice, especially in FGF15^INT-KO^ mice (Figure 4N).

**Figure 5:**
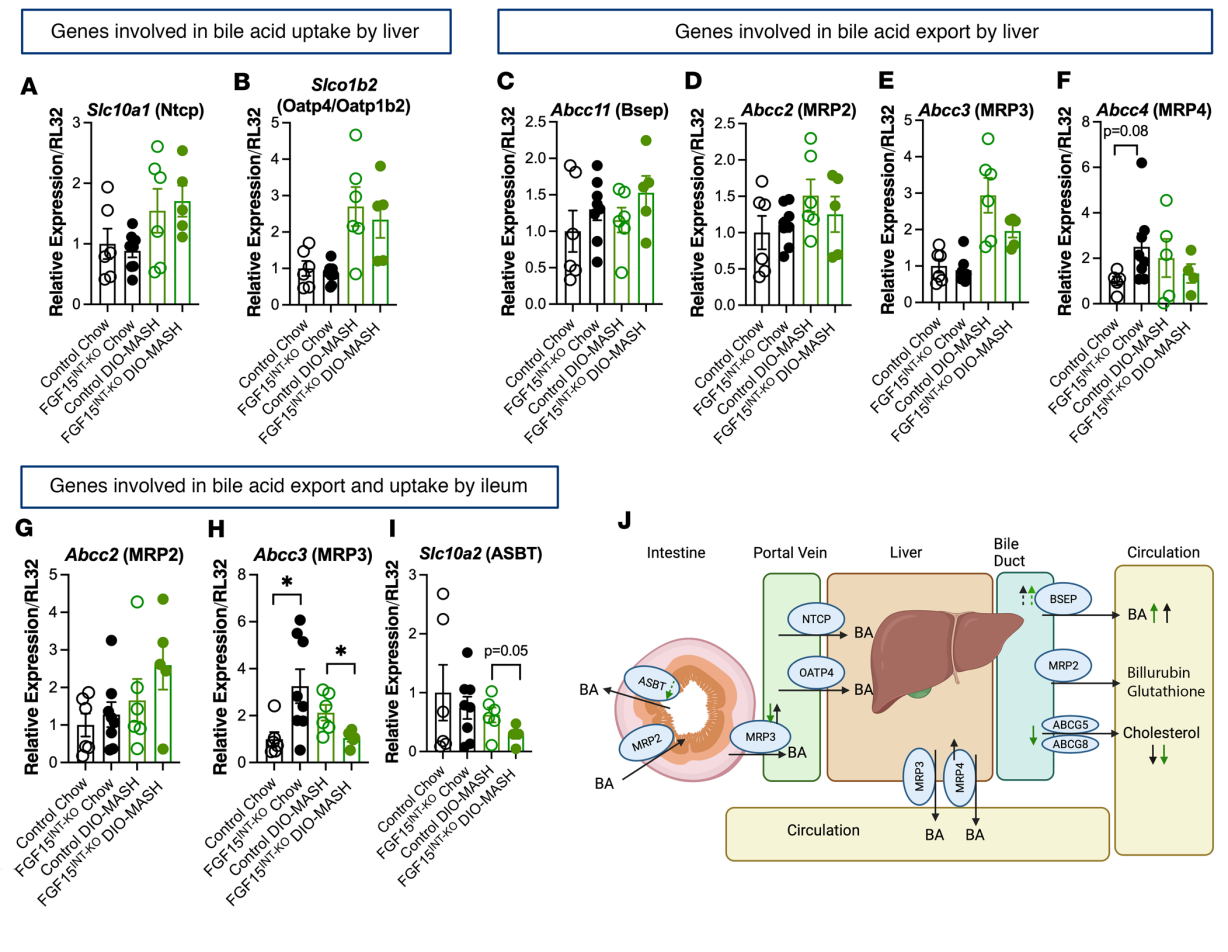
Intestinal FGF15 regulates enterohepatic bile acid metabolism. RNA expression of genes involved in bile acid uptake by liver. (**A**) *Slc10a1* (Ntcp). (**B**) *Slco1b2* (Oatp4). RNA expression of genes involved in bile acid export by liver. (**C**) *Abcc11* (BSEP). (**D**) *Abcc2* (MRP2). (**E**) *Abcc3* (MRP3). (**F**) *Abcc4* (MRP4). RNA expression of genes involved in bile acid export and uptake by ileum. (**G**) *Abcc2* (MRP2). (**H**) *Abcc3* (MRP3). (**I**) *Slc10a2* (ASBT). (**J**) Diagram (adapted from ref. 5) showing the tissue-specific expression of bile acid transporters and how they change in FGF15^INT-KO^ mice compared with controls. Black arrows are for changes in chow diet, and green arrows signify changes in DIO-MASH group. ASBT, apical sodium-dependent bile acid transporter; ABCG5 and ABCG8, hepatic cholesterol efflux pump-ATP-binding cassette, sub-family G, members 5 and 8. Animal numbers for **A**–**E** and **G**–**I** are control chow (*n* = 6), FGF15^INT-KO^ chow (*n* = 8), control DIO-MASH (*n* = 6), FGF15^INT-KO^ DIO-MASH (*n* = 5). Animal numbers for **F** are control chow (*n* = 5), FGF15^INT-KO^ chow (*n* = 8), control DIO-MASH (*n* = 5), FGF15^INT-KO^ DIO-MASH (*n* = 4). Data are shown as means ± SEM. Two-tailed Student’s *t* test (unpaired) comparing responses between genotypes per diet.

Next, we measured the expression of hepatic and ileal bile acid uptake and export transporters. The expression of *Slc10a1* (coding for liver bile acid transporter Ntcp) and *Oatp4* bile acid uptake genes was not different between control and FGF15^INT-KO^ mice (Figure 5, A and B). There was no significant difference in the hepatic expression of genes involved in bile acid export, such as *Abcc11* (coding for bile salt export pump, BSEP), *Abcc2* (coding for multi-drug resistant protein 2, MRP2), and *Abcc3* (coding for multi-drug resistant protein 23, MRP3) (Figure 5, C–E). The gene expression of *Abcc4* (coding for multi-drug resistant protein 4, MRP4) trended to increase in chow-fed FGF15^INT-KO^ mice compared with chow-fed controls but was not different under DIO-MASH diet (Figure 5F). Similarly, the ileal gene expression of *Abcc2* was not different between the genotypes (Figure 5G). However, in the ileum, *Abcc3* showed diet- and genotype-specific expression with increased expression in chow-fed FGF15^INT-KO^ mice compared with controls but decreased expression in DIO-MASH–fed FGF15^INT-KO^ mice compared with controls (Figure 5H). Similarly, we found no difference in the expression of bile acid transporter *Slc10a2* (coding for ASBT) between the genotypes under chow diet, but there was a trend of decreased expression in DIO-MASH FGF15^INT-KO^ mice compared with DIO-MASH controls (Figure 5I). These data suggest that the bile acid flux from enterocytes into portal blood (ileum *Abcc3*) and from hepatocytes into systemic circulation (liver *Abcc4*) are increased in FGF15^INT-KO^ during standard diet to facilitate the increased systemic bile acid levels but decreased on DIO-MASH diet, reflecting the increased cholesterol/bile acid demand (Figure 5J).

### Intestinal FGF15 regulates bile acid synthesis, leading to altered cholesterol levels.

**Figure 6:**
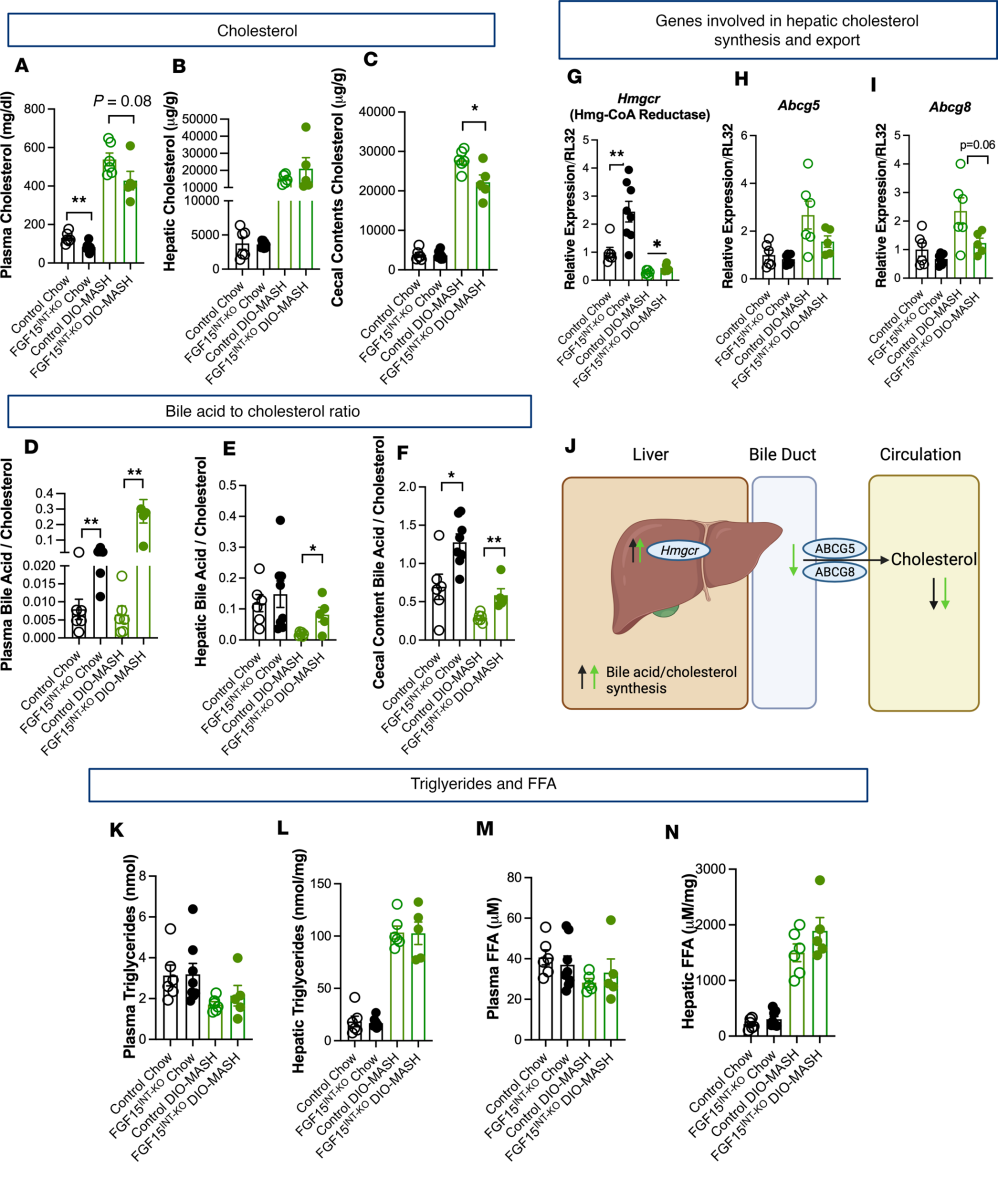
Intestinal FGF15 regulates bile acid synthesis, leading to altered cholesterol levels. (**A**) Plasma cholesterol (postprandial). (**B**) Hepatic cholesterol. (**C**) Cecal content cholesterol. Bile acid to cholesterol ratio in (**D**) plasma, (**E**) liver, and (**F**) cecal contents. Genes involved in hepatic cholesterol synthesis and export (**G**) *Hmgcr* (Hmg-CoA reductase), (**H**) *Abcg5*, and (**I**) *Abcg8*. (**J**) Diagram showing the tissue-specific expression of genes involved in cholesterol synthesis and export and how they change in FGF15^INT-KO^ mice compared with controls. Black arrows are for changes in chow diet, and green arrows signify changes in DIO-MASH group. (**K**) Plasma triglycerides (postprandial). (**L**) Hepatic triglycerides. (**M**) Plasma free fatty acids (FFA; postprandial). (**N**) Hepatic FFA. Animal numbers for **A**–**L** and **N** are control chow (*n* = 6), FGF15^INT-KO^ chow (*n* = 8), control DIO-MASH (*n* = 6), FGF15^INT-KO^ DIO-MASH (*n* = 5). Animal numbers for **M** are control chow (*n* = 6), FGF15^INT-KO^ chow (*n* = 8), control DIO-MASH (*n* = 5), FGF15^INT-KO^ DIO-MASH (*n* = 5). Data are shown as means ± SEM. *P < 0.05, **P < 0.01, 2-tailed Student’s *t* test (unpaired) comparing responses between genotypes per diet.

Bile acids are synthesized from cholesterol in the liver. We examined the tissue cholesterol content in chow and DIO-MASH control and FGF15^INT-KO^ mice. Analysis of tissue-specific lipids revealed lower circulating cholesterol in FGF15^INT-KO^ mice compared with controls when fed chow diet and when fed DIO-MASH diet (Figure 6A). There was no genotype difference in hepatic cholesterol content (Figure 6B). Chow-fed control and FGF15^INT-KO^ mice had comparable levels of cecal content cholesterol levels, but when fed DIO-MASH diet, the FGF15^INT-KO^ mice had lower cholesterol levels in cecal content (Figure 6C). Similarly, the bile acid/cholesterol ratio revealed a higher plasma and cecal content bile acid/cholesterol in FGF15^INT-KO^ mice compared with controls when fed standard chow (Figure 6, D–F). Additionally, the bile acid/cholesterol ratio in mice fed DIO-MASH diet revealed higher plasma, hepatic, and cecal content bile acid/cholesterol in FGF15^INT-KO^ mice compared with controls (Figure 6, D–F). Although the hepatic cholesterol content was not different between FGF15^INT-KO^ and control mice, the hepatic expression of cholesterol synthesis rate limiting gene, 3-hydroxy-3-methyl-glutaryl- coenzyme A reductase, HMG-CoA reductase (*Hmgcr*), was higher in FGF15^INT-KO^ mice (Figure 6G) independent of the diet. Next, we measured the expression of *Abcg5* and *Abcg8*, and found that their expression showed a trend toward a decrease in FGF15^INT-KO^ mice (Figure 6, H and I). These data suggest that despite increased cholesterol synthesis, there is attenuated cholesterol export leading to decreased circulating cholesterol in FGF15^INT-KO^ chow and DIO-MASH mice (Figure 6J). The increased hepatic bile acid/cholesterol ratio in mice fed DIO-MASH (diet that increases hepatic fat and cholesterol content) suggests that the increased cholesterol synthesis is likely directed toward increased bile acid production in the liver (Figure 6J). It is important to note that classical bile acid synthesis genes *Cyp7a1* and *Cyp8b1* are increased in FGF15^INT-KO^ mice regardless of diet (Figure 4, E and F). These data reinforce that lack of FGF15 drives increased bile acid production, which uses up the pool of available cholesterol regardless of diet; however, the diet plays a role in the outcome in plasma and excreted cholesterol levels. Further analysis of circulating and hepatic lipids showed no genotype difference in plasma and hepatic triglycerides (Figure 6, K and L) and plasma and hepatic free fatty acids (FFA) (Figure 6, M and N).

### Intestinal FGF15’s regulation of circulating bile acid levels is not FXR dependent.

**Figure 7:**
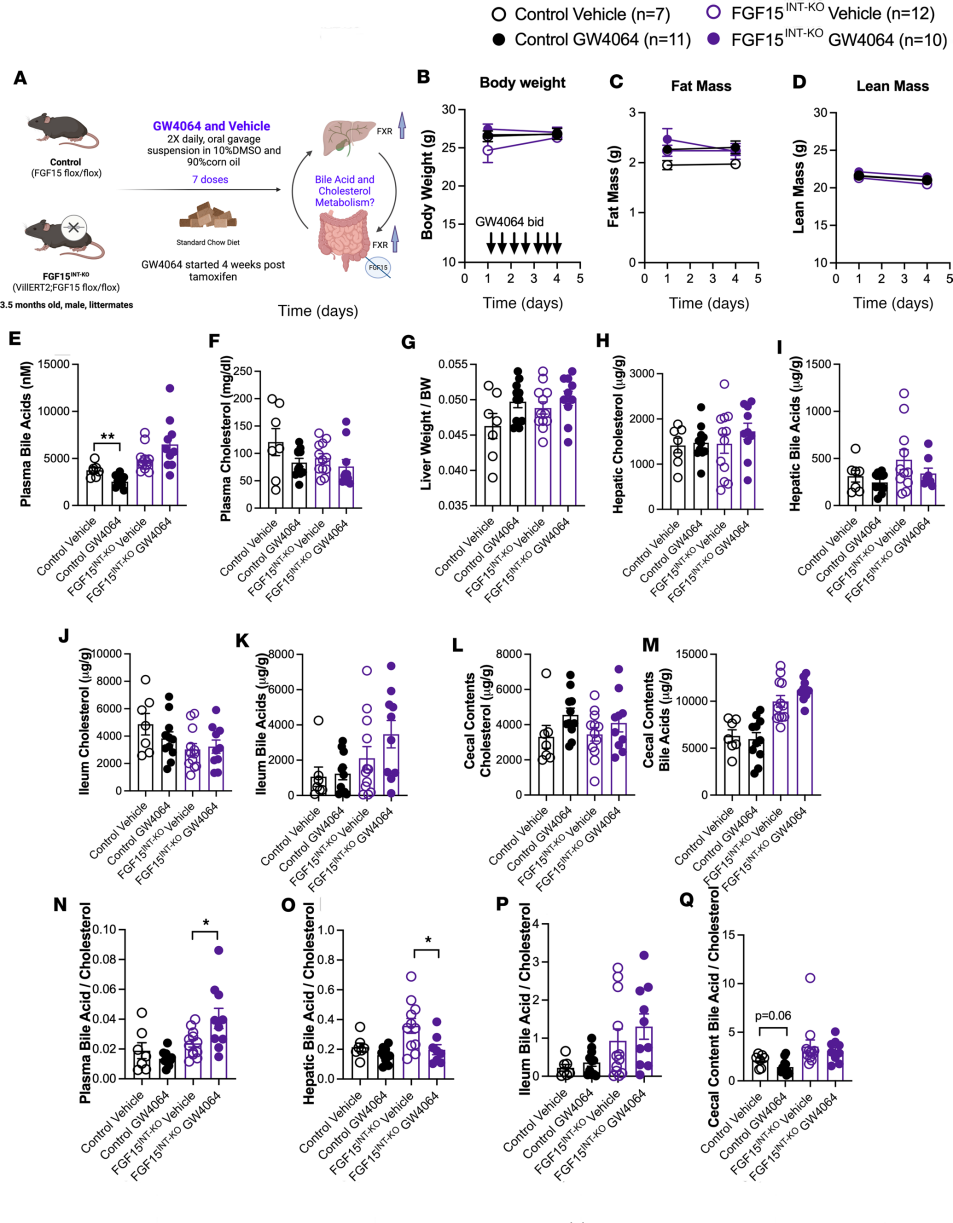
Intestinal FGF15’s regulation of circulating bile acid levels is not FXR dependent. (**A**) Experimental timeline of control and FGF15^INT-KO^ mice fed standard chow diet and administered with FXR agonist GW4064 (50 mg/kg) or vehicle (10% DMSO and 90% corn oil) twice daily for 3 days/total 7 doses. (**B**) Body weight. (**C**) Fat mass. (**D**) Lean mass before and after GW4064/vehicle. (**E**) Plasma total bile acids. (**F**) Plasma cholesterol. (**G**) Liver to body weight ratio. (**H**) Hepatic cholesterol. (**I**) Hepatic total bile acids. (**J**) Ileum cholesterol. (**K**) Ileum total bile acids. (**L**) Cecal contents cholesterol. (**M**) Cecal contents total bile acids. Bile acids to cholesterol ratio in (**N**) Plasma, (**O**) Liver, (**P**) Ileum, and (**Q**) Cecal contents. Animal numbers for **B**–**H**, **J**–**N**, **P**, and **Q** are control vehicle (*n* = 7), control GW4064 (*n* = 11), FGF15^INT-KO^ vehicle (*n* = 12), FGF15^INT-KO^ GW4064 (*n* = 10). Animal numbers for **I** and **O** are control vehicle (*n* = 7), control GW4064 (*n* = 10), FGF15^INT-KO^ vehicle (*n* = 11), FGF15^INT-KO^ GW4064 (*n* = 8). Data are shown as means ± SEM. *P < 0.05, **P < 0.01, 2-tailed Student’s *t* test (unpaired) comparing responses between genotypes per treatment.

Increased expression and secretion of intestinal FGF15/19 is a key response to FXR activation. We asked whether systemic FXR activation can bypass ileum FGF15 in regulation of tissue bile acid levels. We administered synthetic FXR agonist GW4064 and vehicle (50 mg/kg, oral gavage, twice daily) for 7 days to activate FXR in control and FGF15^INT-KO^ mice fed standard chow diet (Figure 7A). GW4064 did not affect body weight, fat mass, or lean mass (Figure 7, B–D). GW4064 administration effectively reduced bile acids, but not cholesterol, in control mice (Figure 7, E and F). However, GW4064 administration did not reduce plasma bile acids or cholesterol in FGF15^INT-KO^ mice (Figure 7, E and F). FXR effectively decreased the hepatic expression of the bile acid synthesis gene *Cyp7a1* in both control and FGF15^INT-KO^ mice (Supplemental Figure 2A). Hepatic expression of *Cyp8b1* was reduced by GW4064 in control but not FGF15^INT-KO^ mice, and no effect of GW4064 was detected in the expression of *Cyp27a1* (Supplemental Figure 2, B and C). Consistent with these findings, GW4064 also increased ileal *FGF15* expression in control mice and showed a trend of decreased ileal *FXR* expression, with no changes in *TGR5* expression (Supplemental Figure 2, F–H). There was no significant change in hepatic bile acid uptake genes *Slc10a1* (Ntcp) and *Slco1b2* (Oatp4) or in bile acid export by liver (*Abcc11*/BSEP) and by ileum (*Slc10a2*/ASBT) by GW4064 in either genotype (Supplemental Figure 2, D, E, I, and J). The hepatic expression of HMG-CoA reductase (*Hmgcr*) was also not different after GW4064 (Supplemental Figure 2K). The expression of hepatic cholesterol efflux *Abcg5* expression and a trend of increased expression in *Abcg8* was observed in both control and FGF15^INT-KO^ mice after GW4064 treatment (Supplemental Figure 2, L and M). Regardless of these transcriptional changes in hepatic bile acid and cholesterol synthesis and transport, GW4064 did not affect the ratio of liver to body weight, hepatic cholesterol, or hepatic bile acids (Figure 7, G–I). Further GW4064 did not induce changes in ileum cholesterol, bile acids (Figure 7, J and K), or cecal content of cholesterol and bile acids (Figure 7, L and M). However, when we analyzed the ratio of plasma bile acid to cholesterol, we observed an increase in FGF15^INT-KO^ + GW4064 mice compared with FGF15^INT-KO^ mice treated with vehicle (Figure 7N). On the contrary, there was a decrease in hepatic bile acid/cholesterol in FGF15^INT-KO^ + GW4064 mice compared with FGF15^INT-KO^ mice treated with vehicle (Figure 7O). There were no differences in ileum bile acid/cholesterol due to GW4064 treatment (Figure 7P). The cecal content bile acid/cholesterol had a trend toward being reduced in control mice but not in FGF15^INT-KO^ mice (Figure 7Q). These data suggest that systemic activation of FXR in the absence of intestinal FGF15 is not sufficient to decrease circulating bile acids but is sufficient to decrease the hepatic bile acid/cholesterol ratio in FGF15^INT-KO^ mice. Therefore, tissue-specific FXR activation has a different physiological role that works in an FGF15-dependent and -independent manner.

### Intestinal FGF15 is not necessary to suppress steatosis and fibrosis in the liver.

**Figure 8:**
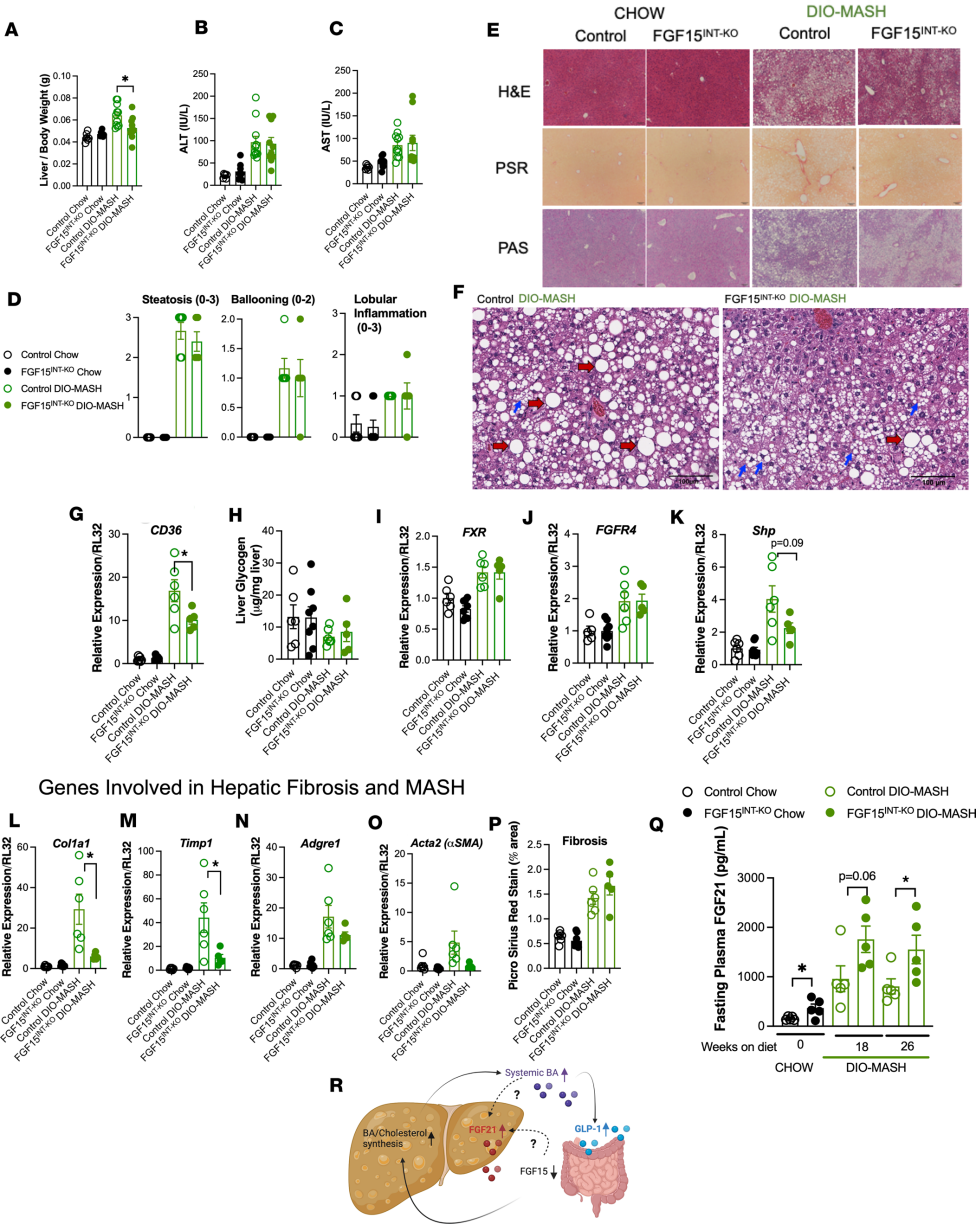
Intestinal FGF15 is not necessary to suppress steatosis and fibrosis in the liver. (**A**) Liver to body weight ratio. (**B**) Alanine aminotransferase (ALT) plasma levels. (**C**) Aspartate aminotransferase (AST) plasma levels. (**D**) Pathology examination of H&E-stained liver (full scan area). (**E**) Representative images of liver stained for H&E, Picro Sirius Red (PSR) for fibrosis analysis, and periodic acid–Schiff (PAS) for glycogen analysis; scale 100 μm. (**F**) Representative images of liver H&E showing macrovesicular (red arrows) and microvesicular (blue arrows) steatosis; scale = 100 μm. (**G**) Liver RNA expression of CD36. (**H**) Liver glycogen content. Liver RNA expression of (**I**) FXR, (**J**) FGFR4, (**K**) small heterodimer partner (SHP), (**L**) Col1a1, (**M**) Timp1, (**N**) Adgre1, and (**O**) Acta2 (α-SMA). (**P**) Analysis of fibrosis by percentage area of PSR stain. (**Q**) Circulating (4-hour fast) FGF21 levels. (**R**) Diagram representing the increased levels of circulating bile acids, GLP-1, and FGF21 in mice lacking intestinal FGF15. Animal numbers for **A** are control chow (*n* = 6), FGF15^INT-KO^ chow (*n* = 7), control DIO-MASH (*n* = 11), FGF15^INT-KO^ DIO-MASH (*n* = 10). Animal numbers for **B** and **C** are control chow (*n* = 6), FGF15^INT-KO^ chow (*n* = 8), control DIO-MASH (*n* = 11), FGF15^INT-KO^ DIO-MASH (*n* = 10). Animal numbers for **D**, **G**–**I**, and **K**–**P** are control chow (*n* = 6), FGF15^INT-KO^ chow (*n* = 8), control DIO-MASH (*n* = 6), FGF15^INT-KO^ DIO-MASH (*n* = 5). Animal numbers for **J** are control chow (*n* = 5), FGF15^INT-KO^ chow (*n* = 8), control DIO-MASH (*n* = 6), FGF15^INT-KO^ DIO-MASH (*n* = 5). Animal numbers for **Q** are control DIO-MASH (*n* = 5), FGF15^INT-KO^ DIO-MASH (*n* = 5). Data are shown as means ± SEM. *P < 0.05, 2-tailed Student’s *t* test (unpaired) comparing responses between genotypes per diet.

Liver/body weight ratios were comparable in the chow-fed groups but lower in DIO-MASH FGF15^INT-KO^ mice compared with DIO-MASH controls (Figure 8A). Although ALT and AST were elevated after DIO-MASH diet, there were no differences between control and FGF15^INT-KO^ mice (Figure 8, B and C). Similarly, histopathologic examination revealed a high degree of steatosis and moderate levels of ballooning and lobular inflammation in DIO-MASH mice, with comparable degrees between controls and FGF15^INT-KO^ mice in both dietary challenges (Figure 8, D and E). Despite the similar level of steatosis, DIO-MASH FGF15^INT-KO^ mice had much higher degrees of microvesicular steatosis compared with macrovesicular steatosis, contrary to the controls, which exhibited robust macrovesicular lipid droplets typically found in patients with MASLD (Figure 8F). The gene expression of key factors involved in hepatic inflammation and lipogenesis, such as *Tnf*, *Ppar*α, *Scd1*, *Cpt1*α, *Srebf1*, and *FAS*, were comparable between genotypes (Supplemental Figure 3, A–F). However, hepatic *CD36* expression was significantly decreased in DIO-MASH FGF15^INT-KO^ mice (Figure 8G). There was also a trend of decreased expression of hepatic *Pdk4* in DIO-MASH FGF15^INT-KO^ mice (Supplemental Figure 3G). These changes in histology were also not related to hepatic glycogen since levels were comparable between the 2 genotypes analyzed by hepatic PAS stain and by measuring hepatic glycogen (Figure 8, E and H). In addition, the expression of gluconeogenesis genes *G6pase* and *Ppargc1* was also comparable between control and FGF15^INT-KO^ mice (Supplemental Figure 3, H and I). The hepatic expression of *Pck1* was decreased in DIO-MASH FGF15^INT-KO^ mice (Supplemental Figure 3J).

Absorbed bile acids activate intestinal FXR and its downstream target FGF15/19. FGF15/19 then enters the portal venous circulation and travels to the liver, where FGF15/19 binds to its receptor, FGFR4, and represses de novo bile acid synthesis and gallbladder filling (8). *FXR* and *FGFR4* gene expression were elevated in DIO-MASH mice but comparable between control and FGF15^INT-KO^ mice (Figure 8, I and J). However, the FXR target *SHP* showed a trend toward reduced expression in DIO-MASH FGF15^INT-KO^ mice compared with DIO-MASH controls (Figure 8K). Next, we focused on elucidating the role of gut FGF15 on hepatic fibrosis induced by DIO-MASH diet. Expression of key fibrosis markers, such as *Col1a1*, *Timp1*, *Adgre1*, and *Acta2*, was increased in response to DIO-MASH diet. However, these markers were either reduced or showed a trend toward reduced expression in DIO-MASH FGF15^INT-KO^ mice compared with controls (Figure 8, L–O). Despite these gene expression differences, the pathological examination of fibrosis with PSR revealed a similar degree of fibrosis between DIO-MASH controls and FGF15^INT-KO^ mice (Figure 8, E and P).

Our previous studies identified increased circulating FGF21 concentration in whole-body *FGF15^–/–^* mice as well as gut-specific FGF15^INT-KO^ mice (24, 42). Circulating FGF21 concentrations were increased in chow-fed FGF15^INT-KO^ mice (Figure 8Q). FGF21 concentration increased with the consumption of DIO-MASH diet but remained elevated in DIO-MASH FGF15^INT-KO^ mice compared with DIO-MASH controls (Figure 8Q). Taken together, these data indicate that similar to increased plasma total GLP1 concentration, plasma FGF21 concentrations are increased in chow and DIO-MASH FGF15^INT-KO^ mice as a compensatory response to the increased bile acid levels and secondary to the lack of intestinal FGF15 (Figure 8R). In the context of MASLD, the question remains whether the decrease in FGF15 precedes and acts as a catalyst for the increase in FGF21 or vice versa.

## Discussion

Consistent with previous work, our data show that postprandial FGF19 concentration is lower in individuals with obesity, without a strong association with postprandial blood glucose levels (28–31). However, our analysis showed that only approximately 20% of the variability in FGF19 is predicted by BMI. Consistent with our findings, a recent study showed that exogenous administration of the FGF19 analog NGM282 did not correct hyperglycemia in patients with type 2 diabetes (T2D) but instead caused a rapid and sustained reduction in hepatic *Cyp7a1* levels and liver fat content in patients with MASLD (36). Further studies and larger cohorts are needed to tease out the association of systemic FGF19 levels and the regulation of body weight and MASLD in humans.

The physiological role of FGF15 in metabolism has been previously described using total-body knockout mice (*FGF15^–/–^*), pharmacological administration of FGF15/19, and mice constitutively overexpressing FGF19. It has been reported that global ablation of FGF15 resulted in impaired glucose tolerance, elevated postprandial hepatic glycogen levels, increased HFD-induced triglycerides, and decreased HFD-induced liver fibrosis (11, 12, 43). While some have reported that *FGF15^–/–^* mice gain more weight and adiposity when challenged with HFD (44), in our hands *FGF15^–/–^* mice were resistant to HFD-induced obesity (42). *FGF19*-transgenic mice were reported as hyperphagic but with lower body weight and fat mass along with increased energy expenditure (17). Peripheral administration or transgenic overexpression of FGF19 has been shown to improve glucose tolerance, suppress hepatic glucose output, and increase hepatic glycogen stores in chow-fed, obese, and leptin-deficient *ob/ob* mice (11, 12, 16, 17, 27).

Circulating FGF15/19 levels are mostly gut derived in the adult mouse and human, and thus the ability to suppress hepatic glucose production and improve glucose tolerance has been attributed to intestine-derived FGF15 (8). However, none of these studies provide clear evidence about the role of FGF15/19 as a gut hormone in the adult animal. Importantly, recent studies have identified that central FGF15-expressing neurons in the DMH control glucagon secretion and hepatic gluconeogenesis, introducing the possibility of a tissue-dependent role of FGF15 (25–27). To directly test the role of gut FGF15 in several metabolic parameters, we used our mouse model of gut-derived FGF15 ablation in the adult mouse (24). The picture that emerged from these comprehensive studies is different from the conclusions drawn from these previous approaches using *FGF15^–/–^* mice. We found that gut-derived FGF15 is not necessary for the regulation of energy balance and glucose metabolism during standard chow and DIO-MASH diets (Figures 2 and 3).

Our findings showed that intestine-derived FGF15 is a critical regulator of bile acid synthesis and bile acid/cholesterol tissue content (Figures 4 and 6). Hepatic *Cyp7a1* was induced significantly in FGF15^INT-KO^ mice, suggesting that the intestine is a major source of circulating FGF15 in mice that is not compensated by FGF15 produced by other tissues. The important role of intestinal FGF15/19 in regulating bile acid production and cholesterol levels highlights the potential for this gut/liver signaling to regulate multiple aspects of liver function that may contribute to disease. MASLD affects up to 25% of the world population and is one of the most prevalent liver diseases (45). MASLD presents with varying phenotypic stages ranging from steatosis to MASH, fibrosis, cirrhosis, and liver carcinoma (45). MASLD and MASH have emerged as leading causes of chronic liver disease that represent a global health challenge. In this study, we challenged mice with DIO-MASH diet, composed of 40% fat (mostly palm oil), 20% fructose, and 2% cholesterol, for 26 weeks. The rationale of choosing this diet also came from our transcriptomic data showing increased expression of genes involved in the fructose and mannose metabolism in FGF15-positive cells isolated from HFD-obese versus lean animals (Figure 1). Previous studies have shown that CCl_4_-induced fibrosis gene expression markers and HFD-induced fibrosis were reduced in total-body *FGF15^–/–^* mice (43, 46). The authors speculated that the increased bile acids in *FGF15^–/–^* mice can activate hepatic FXR and thus attenuate fibrotic development. Our data also showed decreased gene expression of key fibrotic markers in FGF15^INT-KO^ mice compared with the controls fed DIO-MASH diet (Figure 8). However, the pathological examination and quantification of the PSR stain did not reveal differences between the genotypes. It is currently unclear if the attenuated expression of fibrotic markers in FGF15^INT-KO^ DIO-MASH mice is FGF15 dependent or is a result of the increased circulating bile acids or the altered bile acid composition in FGF15^INT-KO^ mice.

Despite having a similar degree of hepatic steatosis, DIO-MASH–fed FGF15^INT-KO^ mice had increased microvesicular and decreased macrovesicular steatosis compared with control DIO-MASH mice (Figure 8). Microvesicular steatosis has been associated with more advanced histology of MASLD and used as an indicator of hepatocellular damage (47, 48). Compared with macrovesicular steatosis, microvesicular steatosis has been suggested to be an independent predictor of advanced liver injury in MASH (48). It has been shown that a defect in fatty acid oxidation results in the accumulation of lipids in the cytosol and the formation of megamitochondria, one of the hallmark features of microvesicular steatosis (47–50). Expression of hepatic *CD36*, a fatty acid transporter correlated with the presence of macrovesicular steatosis, was decreased in DIO-MASH FGF15^INT-KO^ mice. Its deletion has already been shown to be associated with reduced VLDL secretion and increased microvesicular steatosis in obese *ob/ob* mice (51). It is also important to note that microvesicular steatosis is not a common finding in individuals with MASLD and those without chemical or toxin exposure but is one of the pathological findings in intestinal failure–associated liver disease (IFALD) (52). This raises the possibility that FGF15/19-bile acid metabolism is a driver and a potential target for IFALD and gut resection–associated liver injury. Although the mechanism behind these observations is not clear, we observed that DIO-MASH FGF15^INT-KO^ mice also had a trend toward decreased villi height compared with controls (Supplemental Figure 1). Fructose consumption increases villi height to expand the gut surface area and is associated with improved nutrient absorption and adiposity (53). These data suggest that DIO-MASH FGF15^INT-KO^ mice may regulate hepatic steatosis by altering intestinal growth and nutrient absorption when challenged with diet high in fat, fructose, and cholesterol. It is also possible that reduction in FGF15/19 and subsequent changes in bile acid composition alter the gut’s efficiency in lipid absorption and lead to altered lipid processing by the liver. Finally, our studies did not take into account the changes in microbiome composition as a result of altered bile acid pool and the ability of the microbiome to alter the gut lipid absorption.

It has also been previously reported that FGF15/19 stimulates protein and glycogen synthesis, while reducing gluconeogenesis, hepatic triglycerides, and cholesterol (11, 12, 43). Our data showed that intestinal FGF15 does not alter postprandial hepatic glycogen content, hepatic triglycerides, and cholesterol and hepatic gluconeogenesis under standard chow and DIO-MASH diet (Figures 2, 3, 6, and 8, and Supplemental Figure 3). We speculate that the decrease in the liver/body weight ratio of the DIO-MASH FGF15^INT-KO^ compared with the DIO-MASH control mice is a result of the altered fat deposition pattern (increased microvesicular versus macrovesicular steatosis) and not a result of total hepatic triglycerides, cholesterol, and glycogen content. That reduction in liver weight was the contributor to the lower lean muscle mass in DIO-MASH FGF15^INT-KO^ at 26 weeks of diet (Figure 3).

Despite increased hepatic expression of *Hmgcr*, we did not observe increased hepatic cholesterol levels in FGF15^INT-KO^ mice compared to controls in both diets (Figure 6). *Abcg5* and *Abcg8* expression trended to decrease in FGF15^INT-KO^ mice. These data suggest that increased cholesterol synthesis and attenuated cholesterol export in FGF15^INT-KO^ mice are directed toward increased bile acid synthesis and hepatic bile acid content. Under chow conditions, FGF15^INT-KO^ and control mice had comparable bile/acid cholesterol ratio. However, once fed DIO-MASH diet, the FGF15^INT-KO^ mice, unlike controls, did not decrease their hepatic bile acid/cholesterol ratio. The decrease in cholesterol efflux by liver resulted in decreased plasma cholesterol and cecal cholesterol content in FGF15^INT-KO^ mice under both diets (Figure 6). These data show that the synthesis of bile acids is critically dependent on both intestinal FGF15 and the dietary challenge.

A wide range of data previously published and presented here have linked FGF15/19 as a negative feedback signal to restrain bile acid production and limit bile acid levels. Bile acids are transported into enterocytes by *ASBT*, where they can activate *FXR* and promote FGF15/19 gene transcription and secretion (7–9, 34). In addition, bile acids themselves can inhibit further bile acid production via direct actions in the liver mediated by FXR signaling. Therefore, FXR and FGF15 signaling are tightly linked in the regulation of bile acid synthesis and overall hepatic lipid metabolism. We used the potent FXR agonist GW4064 to dissect the roles of FXR (in both gut and liver) versus intestinal FGF15 in the regulation of bile acid synthesis. Our data showed that GW4064 can decrease plasma bile acids in control, but not in FGF15^INT-KO^, mice (Figure 7 and Supplemental Figure 2). This is consistent with published data showing that treatment with the FXR-selective agonist GW4064 significantly repressed *Cyp7a1* in liver-specific FXR-knockout mice, but not gut-derived FXR-knockout mice (54). The authors concluded that the activation of FXR in intestine, but not liver, is required for short-term repression of *Cyp7a1* in liver. They also suggested that this intestine-specific effect of FXR is likely mediated through the induction of FGF15, which is also supported by our data. However, when we analyzed the bile acid to cholesterol ratio, our data showed that GW4064 increased plasma bile acid/cholesterol but decreased hepatic bile acid/cholesterol ratio in FGF15^INT-KO^ mice. This implicates a distinct role for tissue-specific FXR regulation of bile acid and cholesterol metabolism that has both FGF15-dependent and independent actions. We speculate that this may be due to the divergent roles of intestinal versus hepatic FXR agonism in lipid metabolism (37).

FGF15^INT-KO^ mice have higher circulating FGF21 concentration on chow and DIO-MASH diets (Figure 8). Belonging to the FGF19 subfamily, fibroblast growth factor 21 (FGF21) is different from other FGF subfamilies and is mainly involved in controlling metabolism (55, 56). FGF21 is an endocrine FGF that is produced mainly in the liver and adipose tissues, and FGF21 levels are tightly linked to nutritional status. Most importantly, however, clinical and mouse studies have reported increased plasma FGF21 concentration in patients and animal models of MASLD, and thus FGF21 has been proposed as a marker of liver steatosis (57–60). Studies have suggested that the upregulation of FGF21 in MASLD is a protective mechanism in dysregulated metabolic pathways. In fact, FGF21 treatment lowers hepatic fat fraction in patients with MASLD (61). However, it has also been shown that FGF21 acts as a negative regulator of bile acid synthesis. Exogenous FGF21 is a potent suppressor of bile acid synthesis by downregulating *Cyp7a1* and decreasing the total bile acid pool (62). Taken together, these data indicate that plasma FGF21 levels are increased in chow and DIO-MASH FGF15^INT-KO^ mice as a compensatory response to the increased bile acid levels that result from lack of intestinal FGF15. In the background of MASLD, the question remains whether the decrease in FGF15 precedes and acts as a catalyst for the increase in FGF21 or vice versa. Future studies will focus on dissecting the role of elevated FGF21 levels in the FGF15^INT-KO^ mice and its contribution in the hepatic and metabolic outcomes.

In summary, our findings show that gut-derived FGF15 is necessary for the regulation of bile acid and cholesterol levels during standard chow and DIO-MASH diet (high in fat, fructose, and cholesterol). Conversely, gut-derived FGF15 is dispensable for the regulation of energy balance and glucose metabolism. Our study showed that patients with obesity have decreased FGF19 levels. While the decline of FGF15/19 in obesity has been observed as a negative consequence, it is possible that this decline is a compensatory response that increases the hepatic production of bile acids at the expense of cholesterol levels. Consistent with this hypothesis, we find the lack of FGF15 results in decreased plasma and cecal content cholesterol as a result of increased bile acid/cholesterol synthesis and decreased hepatic cholesterol efflux into circulation. The increased bile acid synthesis in FGF15^INT-KO^ mice also results in altered bile acid composition, including the increase in secondary bile acids that may play a role in gut TGR5 signaling and lead to the increased basal GLP-1 concentrations observed in FGF15^INT-KO^ mice fed chow and DIO-MASH diets. Regardless of these positive metabolic effects, FGF15^INT-KO^ mice have neither improved nor impaired energy balance or glucose metabolism, strongly pointing to the lack of gut-derived FGF15’s role in glucose metabolism and energy balance, independent from obesity. However, DIO-MASH FGF15^INT-KO^ mice showed a specific pathology involving the development of microvesicular hepatic steatosis and increased circulating FGF21 levels that should be further evaluated in the development of metabolic liver disease. These results show that intestinal FGF15 plays a major role in bile acid metabolism and the way the gut and liver metabolize lipids when challenged with a diet high in fat, fructose, and cholesterol.

## Methods

### Sex as a biological variable.

Male and female participants were included in the human data. Our study exclusively examined male mice, and it is currently unknown whether the findings are relevant for female mice.

### Human studies.

All participants were enrolled in the Michigan Medicine Weight Management Program and were given an opportunity to opt in to the program’s research component, which was reviewed and approved by the Institutional Review Board of the University of Michigan and registered on ClinicalTrials.gov (NCT02043457). Patients with T2D were diagnosed based on the American Diabetes Association criteria for T2D (A1C ≥ 6.5; fasting blood glucose ≥ 126 mg/dL; or 2-hour blood glucose ≥ 200 mg/dL) or had been previously diagnosed with T2D and were taking glucose-lowering medications for T2D. MASLD was defined based on elevation of ALT and elevated ratio of ALT/AST (1.5×) in the absence of other causes of chronic liver disease. Plasma samples were obtained during an MMTT. After a 12-hour fast, 1 can of Ensure was consumed over 10 minutes along with the simultaneous administration of 650 mg of acetaminophen to estimate gut transit time by measuring serum acetaminophen levels. A catheter was placed in the antecubital vein, and 20 mL of blood was drawn (EDTA plasma tubes 3 × 5 mL and 5 mL serum for acetaminophen and glucose levels) at 0 and 60 minutes and 3 mL drawn at 15, 30, 60, 90, 120, 150, and 180 minutes. Plasma samples at 180 minutes were analyzed for blood glucose (Biosen Glucose Analyzer, EKF Diagnostics) and plasma FGF19 levels (R&D Systems).

### Animals and diet.

The Fgf15^fl/fl^ mice were built using CRISPR/Cas9 technology with *LoxP* sites flanking exon 2 of the FGF15 gene as described previously (24). We bred these mice to VilCreERT2 mice [C57BL background; B6.Cg-Tg(Vil1-cre/ERT2)23Syr/J from Jackson Laboratory] and administered tamoxifen (intraperitoneal, 3 doses total, 48 hours between dose, 150 mg/kg) to VilCreERT2 Fgf15^fl/fl^ and controls (VilCreERT2 and Fgf15^fl/fl^). We validated exon 2 excision within the ileum, where FGF15 is most highly expressed. Male mice (8 weeks of age) were group-housed with ad libitum access to water and food. FGF15 iCre-ERT2 mice were generated by Biocytogen as follows. The stop codon of the FGF15 construct was replaced with a 2A-Cre transgene that results in coexpression of FGF15 tethered to Cre recombinase. The 2A sequence is a self-cleavage motif that breaks the 2 proteins apart awhile they are being made (which makes it specific to FGF15-expressing cells). The targeting construct used BAC DNA as a source for picking up flanking DNA regions that target the 2A-Cre specifically to the stop codon. The targeting construct also contained a selection cassette that allows one to only choose clones that have incorporated the DNA targeting vector. The targeting construct was injected into ES cells (C57BL/6 strain), selection was applied, and then 200 clonal isolates were picked and screened for the correct insertion of the 2A-Cre construct in the genome (screened by Southern blot and PCR). Four clones that had the insertion in the FGF15 gene were identified. Those clones were then injected into blastocysts (BALB/c strain) and implanted in pseudopregnant females. The founders were screened for chimerism and bred, and litters were screened by PCR. The FGF15^iCre-ERT2^ mice were bred to L10^eGFP^ reporter mice (63) and fed standard chow (PicoLab, catalog 5LOD) or 60% HFD (Research Diets Inc, catalog 12492) for 3–4 months. Mice were housed under a 12-hour light/dark cycle in a facility maintained at 25°C with 50%–60% humidity for the duration of the studies.

Following tamoxifen administration, FGF15^INT-KO^ and control male mice (all littermates) were placed on respective diets for the remainder of studies. One cohort of mice (control *n* = 6 and FGF15^INT-KO^ *n* = 8) remained on regular chow diet (PicoLab, catalog 5LOD) for the duration of studies (24 weeks). Two separate cohorts of mice (Cohort 1: Control *n* = 6, FGF15^INT-KO^ *n* = 5; Cohort 2: Control *n* = 5, FGF15^INT-KO^ *n* = 5) were fed DIO-MASH diet (Research Diets Inc, catalog D09100310) containing 40% fat (mostly palm oil), 20% fructose, and 2% cholesterol for 26 weeks. Data were presented from 1 of the 2 cohorts or combined data from both cohorts. Whenever data were combined, the results from each independent cohort were congruent.

At termination of studies, all animals were fasted overnight, administered oral mixed meal (volume 100 μL Ensure Plus, 100 μL 20% Intralipid spiked with a 40 mg dextrose) and sacrificed 2 hours later. Postprandial plasma (collected in lithium heparin-coated microtubes) and tissues were collected and frozen immediately. All animals were euthanized using CO_2_.

### Isolation of ileal FGF15-expressing cells by FACS.

FGF15^iCreERT2^ L10^eGFP^ mice were dosed with tamoxifen (intraperitoneal, 150 mg/kg, every 48 hours for 10 days). Tissues were collected less than 24 hours after last tamoxifen dose. Mice were fasted overnight and re-fed ad lib for 2 hours prior to tissue collection. The small intestine was collected and flushed with cold PBS to remove luminal contents. The ileum was opened and placed into individual tubes containing cold DMEM (high glucose, 4.5 g/L) and processed for cell dissociation (STEMCELL Technologies, 07174). The dissociated intestinal epithelial cells were stained with antibodies against CD31, CD45, and annexin V, and the Sytox Blue, and these cells were presorted with MoFlo Astrios cell sorter (Beckman Coulter) to exclude immune, endothelial, apoptotic, and dead cells. The cells were sorted into lysis buffer (Norgen Biotek 37500) on ice. The cells were flash-frozen and stored at –80°C until RNA-Seq analysis. Intestinal epithelial cells from negative control mice (L10^eGFP^ or WT mouse) were included during gating and FACS. GFP^+^/FGF15^+^ cells represented about 1% of the population (4,000–5,000 cells) as expected. The antibodies used were rat anti-CD45–APC (BioLegend 103111), rat anti-CD31–APC (BioLegend 102409), APC Annexin V apoptosis detection kit with 7-AAD (BioLegend 640930), and Sytox Blue Dead Cell Stain for flow cytometry (Thermo Fisher Scientific S34857). Methodology for RNA library preparation, sequencing, and analysis is included in Supplemental Methods.

### Metabolic studies.

Body weight was monitored biweekly. IPGTT was performed by intraperitoneal injection of 50% dextrose (2 g/kg) in 4-hour fasted mice. MMTT was performed via an oral gavage of liquid meal (volume 200 μL Ensure Plus spiked with a 40 mg dextrose and 4 mg acetaminophen, MilliporeSigma) in 4-hour fasted mice. Blood was obtained from the tail vein, and blood glucose was measured with Biosen Glucose Analyzer (EKF Diagnostics). Blood was collected at baseline and 15 minutes after gavage in EDTA-coated microtubes. Plasma acetaminophen levels were used to assess the rate of gastric emptying and were measured using spectrophotometry assay (Sekisui Diagnostics).

### Statistics.

The statistical analysis for comparisons between the 2 genotypes for each dietary challenge was performed by unpaired (2-tailed) Student’s *t* test. One-way ANOVA (Dunnett’s multiple comparisons test) was used for comparisons between lean human participants and those with obesity, obesity/T2D, and obesity/MASLD. We used multiple linear regression to analyze the relationship of postprandial circulating FGF19 with BMI and blood glucose as independent variables in human subjects. RNA-Seq analysis is described in detail in Supplemental Methods. *P* < 0.05 was considered significant. Statistical analysis was performed using GraphPad Prism 10.0. Data graphs were created using GraphPad Prism 10.0 and R. The ROUT method (Q = 1; Prism 10.0) was used to identify significant outliers. Energy expenditure (EE) data were analyzed using the EE ANCOVA provided by the NIH National Institute of Diabetes and Digestive and Kidney Diseases Mouse Metabolic Phenotyping Centers using their Energy Expenditure Analysis page (http://www.mmpc.org/shared/regression.aspx) and supported by grants DK076169 and DK115255.

### Study approval.

All protocols complied with all relevant ethical regulations for animal and human research. All protocols were approved by the University of Michigan and were in accordance with NIH guidelines. Human participation was voluntary and all participants provided written informed consent.

### Data availability.

All RNA-Seq analysis code and count data can be found at https://github.com/alanrupp/fgf15; commit ID b1e76d4. The RNA-Seq data have been deposited in the Sequence Read Archive and assigned BioProject ID PRJNA993243 (https://www.ncbi.nlm.nih.gov/bioproject/993243). A Supporting Data Values file and supplemental information for all results within the main manuscript and supplemental materials are provided with this paper. Additional methods are included in Supplemental Methods.

## Author contributions

NBK and RJS conceived and designed experiments. NBK, JHS, ZL, NM, SK, AER, CFB, and NS performed the experiments and analyzed results. NL and PH performed liver pathology examination. AM, OM, RWO, and RK helped with data interpretation and discussion. NBK, JHS, ZL, ACR, NM, SK, NL, PH, NS, AM, OAM, RWO, RK, CFB, AER, and RJS edited the manuscript. NBK wrote the manuscript and provided final approval of the submitted manuscript.

## Supplementary Material

Supplemental data

Supporting data values

## Version 1 04/08/2024

Electronic publication

## References

[1] Afshin A (2017). Health effects of overweight and obesity in 195 countries. N Engl J Med.

[2] Khan MAB (2020). Epidemiology of type 2 diabetes - global burden of disease and forecasted trends. J Epidemiol Glob Health.

[3] Majait S (2023). The black box orchestra of gut bacteria and bile acids: who is the conductor?. Int J Mol Sci.

[4] Chiang JY (2013). Bile acid metabolism and signaling. Compr Physiol.

[5] Perino A (2021). Molecular physiology of bile acid signaling in health, disease, and aging. Physiol Rev.

[6] Chiang JYL, Ferrell JM (2020). Bile acid receptors FXR and TGR5 signaling in fatty liver diseases and therapy. Am J Physiol Gastrointest Liver Physiol.

[7] Fon Tacer K (2010). Research resource: comprehensive expression atlas of the fibroblast growth factor system in adult mouse. Mol Endocrinol.

[8] Inagaki T (2005). Fibroblast growth factor 15 functions as an enterohepatic signal to regulate bile acid homeostasis. Cell Metab.

[9] Somm E, Jornayvaz FR (2018). Fibroblast growth factor 15/19: from basic functions to therapeutic perspectives. Endocr Rev.

[10] Kliewer SA, Mangelsdorf DJ (2015). Bile acids as hormones: the FXR-FGF15/19 pathway. Dig Dis.

[11] Kir S (2011). FGF19 as a postprandial, insulin-independent activator of hepatic protein and glycogen synthesis. Science.

[12] Potthoff MJ (2011). FGF15/19 regulates hepatic glucose metabolism by inhibiting the CREB-PGC-1α pathway. Cell Metab.

[13] Gimeno L (2003). Study of Fgf15 gene expression in developing mouse brain. Gene Expr Patterns.

[14] Gimeno L (2002). Analysis of Fgf15 expression pattern in the mouse neural tube. Brain Res Bull.

[15] McWhirter JR (1997). A novel fibroblast growth factor gene expressed in the developing nervous system is a downstream target of the chimeric homeodomain oncoprotein E2A-Pbx1. Development.

[16] Fu L (2004). Fibroblast growth factor 19 increases metabolic rate and reverses dietary and leptin-deficient diabetes. Endocrinology.

[17] Tomlinson E (2002). Transgenic mice expressing human fibroblast growth factor-19 display increased metabolic rate and decreased adiposity. Endocrinology.

[18] Morton GJ (2013). FGF19 action in the brain induces insulin-independent glucose lowering. J Clin Invest.

[19] Ryan KK (2013). Fibroblast growth factor-19 action in the brain reduces food intake and body weight and improves glucose tolerance in male rats. Endocrinology.

[20] Miyata M (2011). Fibroblast growth factor 19 treatment ameliorates disruption of hepatic lipid metabolism in farnesoid X receptor (Fxr)-null mice. Biol Pharm Bull.

[21] Kim YC (2020). Intestinal FGF15/19 physiologically repress hepatic lipogenesis in the late fed-state by activating SHP and DNMT3A. Nat Commun.

[22] Angelin B (2012). Circulating fibroblast growth factors as metabolic regulators--a critical appraisal. Cell Metab.

[23] Montagnani M (2011). A new model for portal protein profile analysis in course of ileal intraluminal bile acid infusion using an in situ perfused rat intestine. Med Chem.

[24] Bozadjieva-Kramer N (2021). Intestinal-derived FGF15 protects against deleterious effects of vertical sleeve gastrectomy in mice. Nat Commun.

[25] Picard A (2016). A genetic screen identifies hypothalamic Fgf15 as a regulator of glucagon secretion. Cell Rep.

[26] Picard A (2021). Fgf15 neurons of the dorsomedial hypothalamus control glucagon secretion and hepatic gluconeogenesis. Diabetes.

[27] Lan T (2017). FGF19, FGF21, and an FGFR1/beta-Klotho-activating antibody act on the nervous system to regulate body weight and glycemia. Cell Metab.

[28] Mraz M (2011). Serum concentrations of fibroblast growth factor 19 in patients with obesity and type 2 diabetes mellitus: the influence of acute hyperinsulinemia, very-low calorie diet and PPAR-α agonist treatment. Physiol Res.

[29] Gallego-Escuredo JM (2015). Opposite alterations in FGF21 and FGF19 levels and disturbed expression of the receptor machinery for endocrine FGFs in obese patients. Int J Obes (Lond).

[30] Gomez-Ambrosi J (2017). FGF19 and FGF21 serum concentrations in human obesity and type 2 diabetes behave differently after diet- or surgically-induced weight loss. Clin Nutr.

[31] Barutcuoglu B (2011). Fibroblast growth factor-19 levels in type 2 diabetic patients with metabolic syndrome. Ann Clin Lab Sci.

[32] Eren F (2012). Preliminary evidence of a reduced serum level of fibroblast growth factor 19 in patients with biopsy-proven nonalcoholic fatty liver disease. Clin Biochem.

[33] Jiao N (2018). Suppressed hepatic bile acid signalling despite elevated production of primary and secondary bile acids in NAFLD. Gut.

[34] Morton GJ (2014). Carbohydrate feeding dissociates the postprandial FGF19 response from circulating bile acid levels in humans. J Clin Endocrinol Metab.

[35] Harrison SA (2018). NGM282 for treatment of non-alcoholic steatohepatitis: a multicentre, randomised, double-blind, placebo-controlled, phase 2 trial. Lancet.

[36] DePaoli AM (2019). FGF19 analog as a surgical factor mimetic that contributes to metabolic effects beyond glucose homeostasis. Diabetes.

[37] Bozadjieva N (2018). Targeting FXR and FGF19 to treat metabolic diseases-lessons learned from bariatric surgery. Diabetes.

[38] Harrison SA (2020). NGM282 improves liver fibrosis and histology in 12 weeks in patients with nonalcoholic steatohepatitis. Hepatology.

[39] Haluzikova D (2013). Laparoscopic sleeve gastrectomy differentially affects serum concentrations of FGF-19 and FGF-21 in morbidly obese subjects. Obesity (Silver Spring).

[40] Daryadel A (2019). Elevated FGF23 and disordered renal mineral handling with reduced bone mineralization in chronically erythropoietin over-expressing transgenic mice. Sci Rep.

[41] Duan Y (2019). Hepatic cholesterol accumulation ascribed to the activation of ileum Fxr-Fgf15 pathway inhibiting hepatic Cyp7a1 in high-fat diet-induced obesity rats. Life Sci.

[42] Myronovych A (2020). Assessment of the role of FGF15 in mediating the metabolic outcomes of murine vertical sleeve gastrectomy (VSG). Am J Physiol Gastrointest Liver Physiol.

[43] Schumacher JD (2017). The effect of fibroblast growth factor 15 deficiency on the development of high fat diet induced non-alcoholic steatohepatitis. Toxicol Appl Pharmacol.

[44] Alvarez-Sola G (2017). Fibroblast growth factor 15/19 (FGF15/19) protects from diet-induced hepatic steatosis: development of an FGF19-based chimeric molecule to promote fatty liver regeneration. Gut.

[45] Altavela JL (2017). Population health management: An independent physician organization approach. Am J Health Syst Pharm.

[46] Schumacher JD (2020). Direct and indirect effects of fibroblast growth factor (FGF) 15 and FGF19 on liver fibrosis development. Hepatology.

[47] Tandra S (2011). Presence and significance of microvesicular steatosis in nonalcoholic fatty liver disease. J Hepatol.

[48] Celebi G (2020). Microvesicular steatosis: a missed item in the management of nonalcoholic fatty liver disease?. Acta Gastroenterol Belg.

[49] Ikura Y (2006). Localization of oxidized phosphatidylcholine in nonalcoholic fatty liver disease: impact on disease progression. Hepatology.

[50] Germano CW (2023). Microvesicular steatosis in individuals with obesity: a histological marker of non-alcoholic fatty liver disease severity. Obes Surg.

[51] Nassir F (2013). CD36 deletion reduces VLDL secretion, modulates liver prostaglandins, and exacerbates hepatic steatosis in ob/ob mice. J Lipid Res.

[52] Buchman AL (2017). The differentiation of intestinal-failure-associated liver disease from nonalcoholic fatty liver and nonalcoholic steatohepatitis. Semin Liver Dis.

[53] Taylor SR (2021). Dietary fructose improves intestinal cell survival and nutrient absorption. Nature.

[54] Kim I (2007). Differential regulation of bile acid homeostasis by the farnesoid X receptor in liver and intestine. J Lipid Res.

[55] Itoh N (2010). Hormone-like (endocrine) Fgfs: their evolutionary history and roles in development, metabolism, and disease. Cell Tissue Res.

[56] Henriksson E, Andersen B (2020). FGF19 and FGF21 for the treatment of NASH-two sides of the same coin? Differential and overlapping effects of FGF19 and FGF21 from mice to human. Front Endocrinol (Lausanne).

[57] Dushay J (2010). Increased fibroblast growth factor 21 in obesity and nonalcoholic fatty liver disease. Gastroenterology.

[58] Zhang X (2008). Serum FGF21 levels are increased in obesity and are independently associated with the metabolic syndrome in humans. Diabetes.

[59] Li H (2010). Fibroblast growth factor 21 levels are increased in nonalcoholic fatty liver disease patients and are correlated with hepatic triglyceride. J Hepatol.

[60] Yilmaz Y (2010). Increased serum FGF21 levels in patients with nonalcoholic fatty liver disease. Eur J Clin Invest.

[61] Harrison SA (2021). Efruxifermin in non-alcoholic steatohepatitis: a randomized, double-blind, placebo-controlled, phase 2a trial. Nat Med.

[62] Chen MM (2018). FGF21 acts as a negative regulator of bile acid synthesis. J Endocrinol.

[63] Allison MB (2018). Defining the transcriptional targets of leptin reveals a role for Atf3 in leptin action. Diabetes.

