# Supplementary material for: Intestinal FGF15 regulates bile acid and cholesterol metabolism but not glucose and energy balance
Source: JCI Insight. 2024 Apr 8;9(7):e174164. doi: 10.1172/jci.insight.174164 (PMC11128213; doi:10.1172/jci.insight.174164)
Supplement: Supplemental data [file jciinsight-9-174164-s199.pdf]

SUPPLEMENTAL FIGURES

Supplemental Figure 1

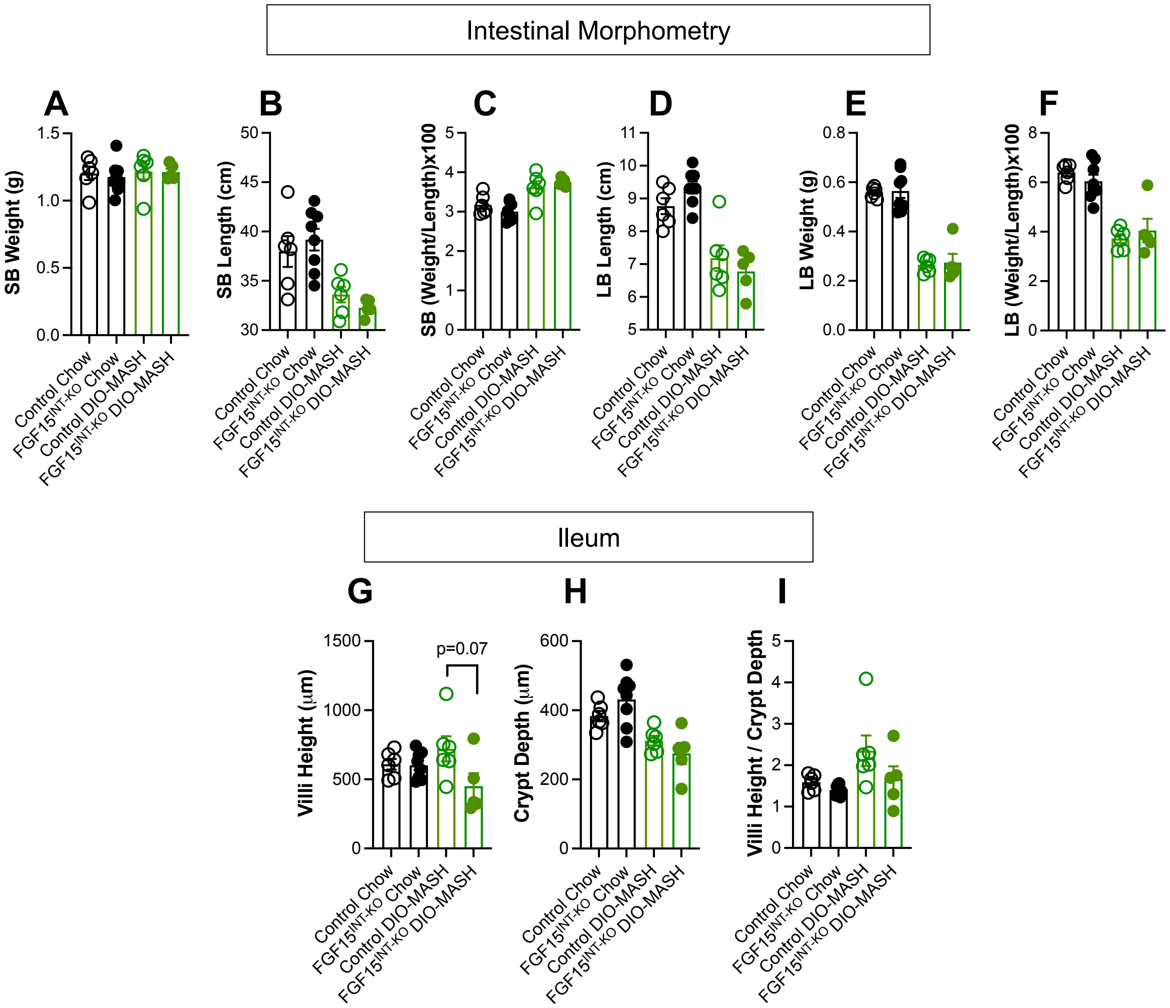

Supplemental Figure 1. Intestial morphometry is not dependent on intestinal FGF15 expression. SB: small bowel, LB: large bowel. **A.** SB weight. **B.** SB length. **C.** SB weight/length. **D.** LB length. **E.** LB weight. **F.** LB weight/length. **G.** Ileum villi height. **H.** Ileum crypt depth. **I.** Ileum villi height/crypt depth. Animal number: control chow (n=6), $FGF15^{INT-KO}$ chow (n=8), control DIO-MASH (n=6), $FGF15^{INT-KO}$ DIO-MASH (n=5). Data are shown as means ± S.E.M. *$p<0.05$, two-tailed Student's *t* test (unpaired) comparing responses between genotypes per diet.

Supplemental Figure 2

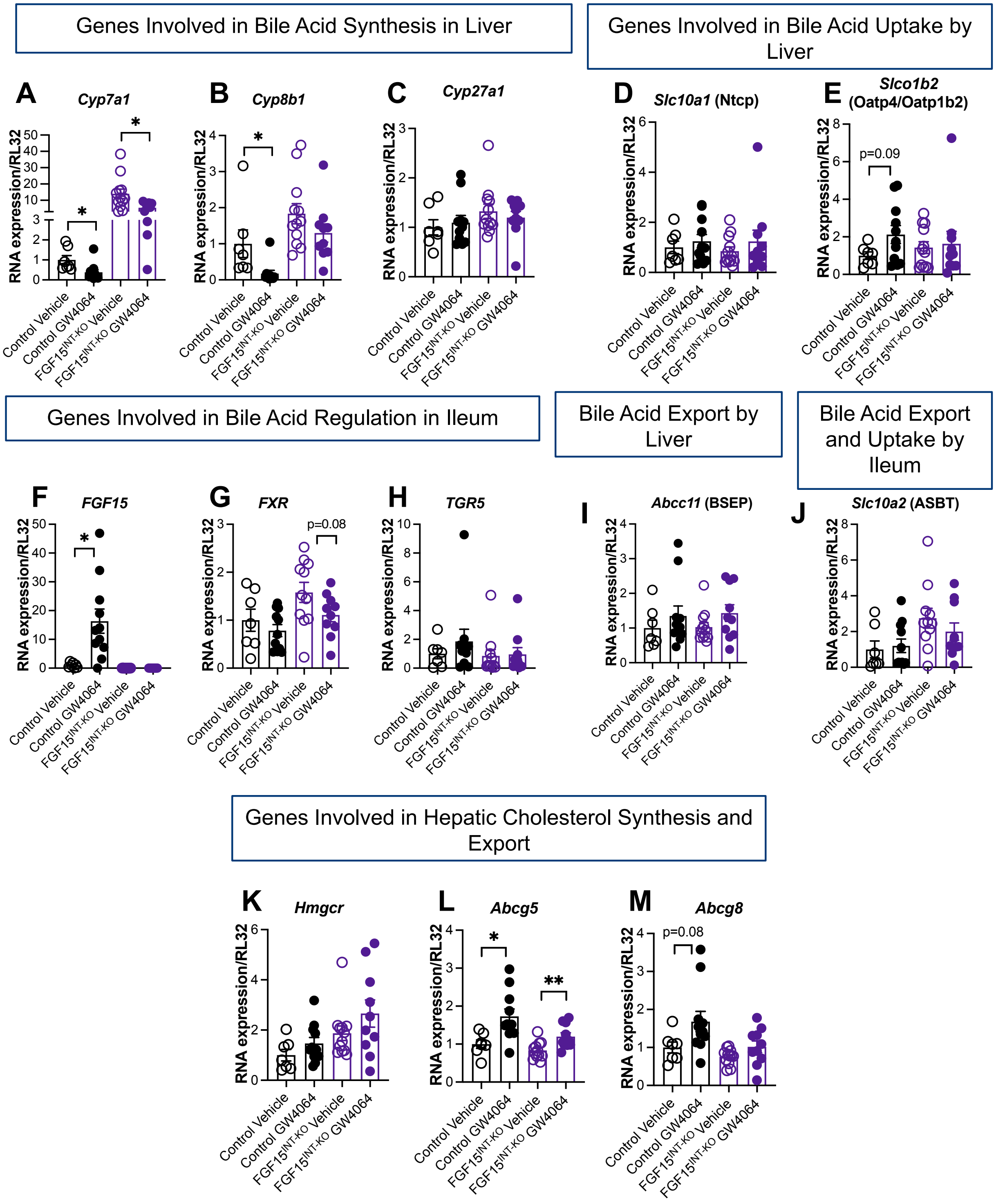

Supplemental Figure 2. Genes involved in hepatic and gut bile acid and cholesterol synthesis and transport after GW4064 treatment in control and FGF15$^{INT\text{-}KO}$ mice. Liver RNA expression of **A.** *Cyp7a1*, **B.** *Cyp8b1*, **C.** *Cyp27a1*, **D.** *Slc10a1* (Ntcp), **E.** *Slco1b2* (Oatp4). Ileal RNA expression of **F.** *FGF15*, **G.** *NR1H4* (FXR) and **H.** *GPBAR1* (TGR5). Liver RNA expression of **I.** *Abcc11* (BSEP). Ileal RNA expression of **J.** *Slc10a2* (ASBT). Liver RNA expression of **K.** *Hmgcr*, **L.** *Abcg5* and **M.** *Abcg8.* Animal numbers for A-E, I, K-M are control vehicle (n=7), control GW4064 (n=11), FGF15$^{INT\text{-}KO}$ vehicle (n=12), FGF15$^{INT\text{-}KO}$ GW4064 (n=10). Animal numbers for F, G and J are control vehicle (n=7), control GW4064 (n=11), FGF15$^{INT\text{-}KO}$ vehicle (n=11), FGF15$^{INT\text{-}KO}$ GW4064 (n=10). Animal numbers for H are control vehicle (n=7), control GW4064 (n=10), FGF15$^{INT\text{-}KO}$ vehicle (n=11), FGF15$^{INT\text{-}KO}$ GW4064 (n=10). Data are shown as means ± S.E.M. *$p<0.05$, two-tailed Student's *t* test (unpaired) comparing responses between genotypes per treatment.

Supplemental Figure 3

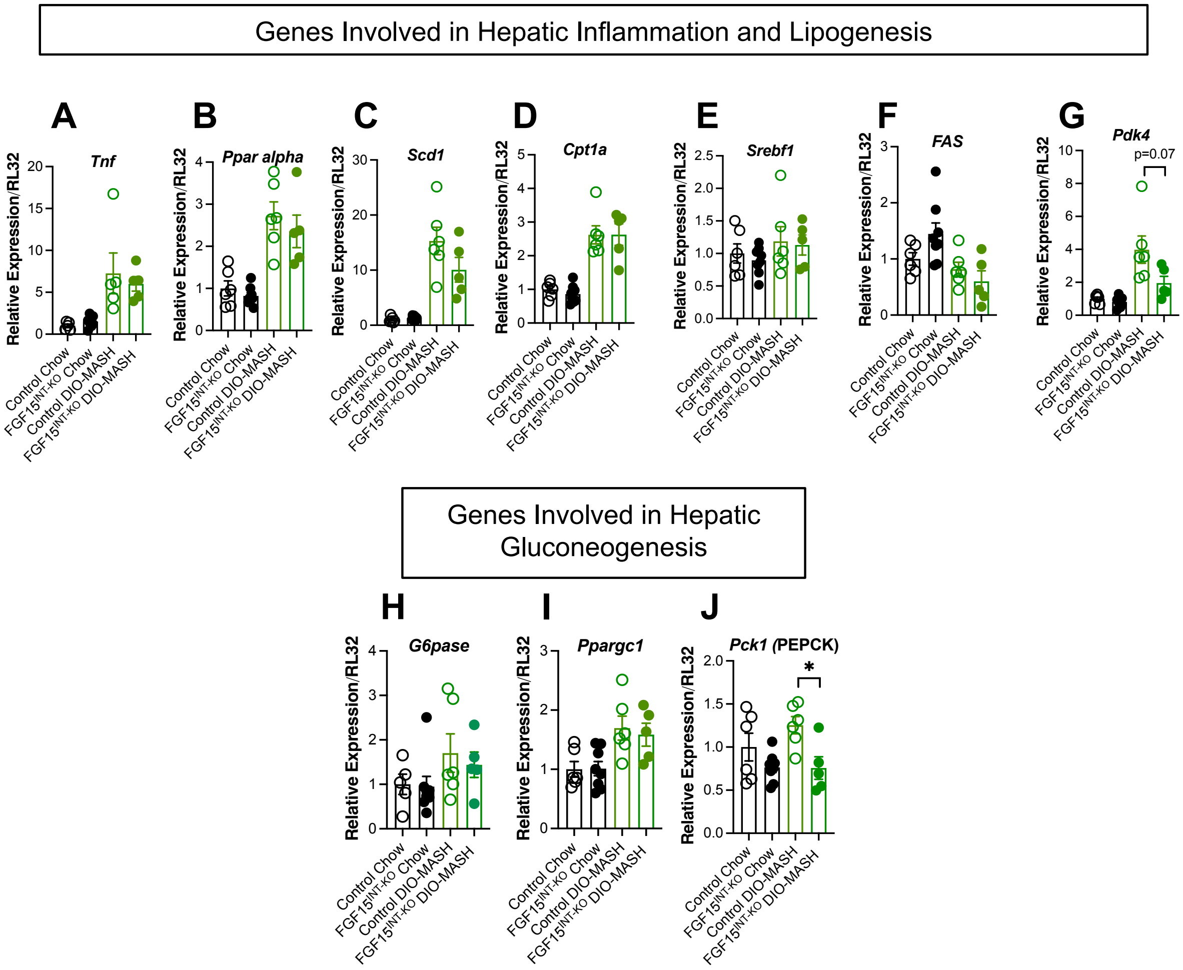

Supplemental Figure 3. Genes involved in hepatic inflammation, lipogenesis and gluconeogenesis. Liver RNA expression of genes involved in hepatic inflammation and lipogenesis, **A.** *Tnf*, **B.** *Ppar alpha*, **C.** *Scd1*, **D.** *Cpt1α*, **E.** *Srebf1*. **F.** *FAS,* **G.** *Pdk4*. Liver RNA expression of genes involved in hepatic gluconeogenesis, **H.** *G6pase*, **I.** *Ppargc1*, **J.** *Pck1* (PEPCK). Animal number for A is control chow (n=5), FGF15^{INT-KO} chow (n=8), control DIO-MASH (n=5), FGF15^{INT-KO} DIO-MASH (n=5). Animal numbers for B-G and I, J are control chow (n=6), FGF15^{INT-KO} chow (n=8), control DIO-MASH (n=6), FGF15^{INT-KO} DIO-MASH (n=5). Animal numbers for H are control chow (n=5), FGF15^{INT-KO} chow (n=8), control DIO-MASH (n=6), FGF15^{INT-KO} DIO-MASH (n=5). Data are shown as means ± S.E.M. *p<0.05, two-tailed Student's *t* test (unpaired) comparing responses between genotypes per diet.

SUPPLEMENTAL METHODS

Metabolite Assays: Insulin (Crystal Chem), total GLP-1 (MesoScale Discovery) and acetaminophen (Sekisui Diagnostics) were measured during MMTT. Postprandial plasma obtained at termination of studies (see above for details) was used to measure FGF23 (Abcam). Glycogen content was measured in liver samples with Glycogen Assay kit (Cayman Chemical). All assays were performed according to the manufacturer's instructions. Fasting plasma (4-hour fast, collected in EDTA-coated microtubes) was collected at 0 (chow diet), 18 and 26 weeks in the cohort fed DIO-MASH diet for the analysis of FGF21 levels.

Lipid and Bile Acid Assays: Liver and cecal content lipids were extracted using Lipid Extraction Kit Chloroform Free (Abcam). Total cholesterol (Pointe Scientific), triglycerides (Abcam), free fatty acids (Abcam) and total bile acids (Total Bile Assay (NBT Method), GenWay Biotech Inc) were measured using the extracted lipids. Postprandial plasma obtained at termination of studies (see above for details) was used to measure total cholesterol (Pointe Scientific), triglycerides (Abcam), free fatty acids (Abcam), ALT and AST (Pointe Scientific). All assays were performed according to the manufacturer's instructions. Plasma bile acid composition was measured in postprandial, terminal plasma by the University of Michigan Metabolomics (see Supplemental Information).

Indirect Gas Calorimetry and Body Composition: Body composition was measured using an EchoMRI (Echo Medical Systems). Indirect gas calorimetry (animal's oxygen consumption (VO2) and carbon dioxide production (VCO2) to estimate various metabolic parameters, including the respiratory exchange rate (RQ), energy expenditure, substrate utilization, food and liquid intake, and locomotor activity were measured using 24-cage TSE PhenoMaster system (TSE Systems; Germany). Mice were allowed to adjust to the metabolic cage for 3 days and measurements were recorded for the following 3 days and averaged for the results. Energy expenditure (EE) data were analyzed using the EE analysis of covariance (ANCOVA) analysis provided by the NIDDK Mouse Metabolic Phenotyping Centers (MMPC, www.mmpc.org) using their Energy Expenditure Analysis page (http://www.mmpc.org/shared/regression.aspx) and supported by grants DK076169 and DK115255.

RNA Library Preparation and Sequencing: Total RNA was isolated from GFP+ (FGF15+) and GFP- cells. RNA libraries were prepared with SMARTer Stranded Total RNA v2 Pico (Takara Bio Inc. #634417) and run on the Illumina HiSeq 4000 as single-end 50 cycles. Library prep and next-generation sequencing were carried out in the Advanced Genomics Core at the University of Michigan.

RNASeq Analysis: FASTQ files were subjected to quality control with FASTQC and low-quality reads (phred < 20) were removed with FASTX-Toolkit. Files were then mapped to a custom mouse genome (GRCm39) containing appended sequences for CreERT2 and EGFP:L10a using STAR (2.7.2a) and gene-level counts were calculated

with the `--quantMode GeneCounts` flag. Resulting count matrices were analyzed in R (4.2.2). Of the 18 count matrices, 2 were removed due to very low read depth (<5 M mapped reads) and/or poor expression correlation and clustering with other samples. HFD-regulated genes were identified using DESeq2 (1.38.3). The HFD-regulated gene list was subjected to KEGG pathway analysis using cluster Profiler (4.6.2). All analysis code and count data can be found at https://github.com/alanrupp/fgf15. The data have been deposited in the Sequence Read Archive (SRA) where they have been assigned BioProject ID PRJNA993243 (https://www.ncbi.nlm.nih.gov/bioproject/993243).

Plasma Bile Acid Composition: Plasma bile acid composition was measured by the University of Michigan Metabolomics Core using two-step solvent extraction. Supernatants were combined, dried, and re-suspended for LCMS separation by RPLC and measurements by ESI^- QQQ MRM methods[1]. *Sample preparation*: 20 µL plasma was transferred to a microtube. 80 µL of chilled acetonitrile with 5% NH4OH, containing isotope labeled internal standards was added to the tube, and the mixture vortexed until completely homogenized. The mixture was incubated on ice for 10 minutes and vortexed to remix. This homogenized mixture was centrifuged and 3 µL of the supernatant from each sample was removed to create a pooled sample for QC purposes. Next, 90 µL of the supernatant was transferred to an LC-MS autosampler insert and brought to dryness in a speedvac set to 45 °C for approximately 45 minutes. Each sample was reconstituted in 100 µL of 50/50 Methanol/Water. A series of calibration standards ranging from 0 to 1000 nM were prepared along with samples to quantify metabolites. *LC-MS analysis*: LC-MS analysis was performed on an Agilent system consisting of a 1290 UPLC module coupled with a 6490 Triple Quad (QqQ) mass spectrometer (Agilent Technologies, Santa Clara, CA) operated in MRM mode. Metabolites were separated on a 100mm x 2.1mm Acquity BEH UPLC (1.7 µm) column (Waters Corp, Milford, MA) using H_2O, 0.1% Formic acid, as mobile phase A, and Acetonitrile, 0.1% Formic acid, as mobile phase B. The flow rate was 0.25 mL/min with the following gradient: linear from 5 to 25% B over 2 minutes, linear from 25 to 40% B over 14 mins, linear from 40 to 95% B over 2 minutes, followed by isocratic elution at 95% B for 5 minutes. The system was returned to starting conditions (5% B) in 0.1 min and held there for 3 minutes to allow for column re-equilibration before injecting another sample. The mass spectrometer was operated in ESI- mode according to previously published conditions. Data were processed using MassHunter Quantitative analysis version B.07.00. Metabolites were normalized to the nearest isotope labeled internal standard and quantitated using 2 replicated injections of 5 standards to create a linear calibration curve with accuracy better than 80% for each standard. Using ROUT method (Q=1), we identified and excluded significant outliers in all four groups.

Bone Parameters: Tissues were fixed in 10% neutral-buffered formalin for 24 hours and kept in Sorenson's buffer (pH7.4) thereafter. Tibiae were placed in a 19-mm diameter specimen holder and scanned over the entire length of the tibiae using a microcomputed tomography (µCT) system (µCT100 Scanco Medical). Scan settings were as follows: voxel size 12 µm, 70 kVp, 114 µA, 0.5 mm AL filter, and integration

time 500 ms. Density measurements were calibrated to the manufacturer's hydroxyapatite phantom. Analysis was performed using the manufacturer's evaluation software, setting a threshold of 180 for trabecular bone and 280 for cortical bone. Tibiae used for µCT scanning were decalcified in 14% EDTA for 3 weeks.

Intestinal Biometry: Following euthanasia, the entire gastrointestinal tract from the stomach to the rectum was removed, cleaned of mesenteric fat and gut weight and length determined. Small and large intestine/colon length was measured on a horizontal ruler after flushing with PBS. The entire small and large intestine/colon were then blotted to remove PBS before being weighed. Ileal sections were dissected and fixed in 10% neutral buffered formalin overnight. Tissue was embedded in paraffin and sectioned onto slides and stained for H&E following standard protocol. Photos and analysis of ileal crypt depth and ileal villi height were acquired using Olympus IX73 fluorescence microscopy system (Olympus). Villus height was measured from the crypt-villus junction to the tip of the villus and crypt depth was measured from the base of the crypt to the crypt-villus junction. Images were analyzed using open-source software ImageJ (imagej.nih.gov).

Liver Histology: Following euthanasia, a liver lobe was removed and fixed in 10% normal buffered formalin overnight. Tissue was embedded in paraffin, sectioned and stained for H&E, Picro Sirius Red (Abcam kit) and Periodic Acid-Schiff stain (PAS) stain. Stains for H&E and PAS were done by University of Michigan In-Vivo Animal Core (IVAC). Picro Sirius Red (PSR) stain was used to measure the degree of fibrosis by quantifying the average percent stain area in approximately 10 sections (10X) of DIO-MASH livers and 2-3 (10X) sections of chow-fed mice livers using open-source software ImageJ (imagej.nih.gov).

Quantitative Real-Time PCR: RNA was extracted from tissue samples using PureLink RNA Mini Kit (Invitrogen). cDNA was synthesized by reverse transcription from mRNA using the iScript cDNA Synthesis Kit (Bio-Rad). Gene expression was performed by quantitative real time RT-PCR using Taqman gene expression assay and was performed using StepOnePlus detection system (Applied Biosystems) with a standard protocol. Relative abundance for each transcript was calculated by a standard curve of cycle thresholds and normalized to RL32.

References:

1. Griffiths WJ, Sjovall J. Bile acids: analysis in biological fluids and tissues. J Lipid Res 2010;51:23-41.
